# A new versatile peroxidase with extremophilic traits over-produced in MicroTom cell cultures

**DOI:** 10.1038/s41598-023-42597-x

**Published:** 2023-09-15

**Authors:** Marta Gogliettino, Ennio Cocca, Fabio Apone, Sonia Del Prete, Marco Balestrieri, Sara Mirino, Stefania Arciello, Gianna Palmieri

**Affiliations:** 1 Institute of Biosciences and BioResources, National Research Council, Via Pietro Castellino 111, 80131 Naples, Italy; 2 Novamont SpA, loc. La Fagianeria Snc, Piana di Monte Verna, Caserta, Italy; 3 Arterra Bioscience SpA, via B. Brin 69, 80142 Naples, Italy

**Keywords:** Biochemistry, Plant sciences, Plant cell biology

## Abstract

Peroxidases are widespread key antioxidant enzymes that catalyse the oxidation of electron donor substrates in parallel with the decomposition of H_2_O_2_. In this work, a novel tomato peroxidase, named SAAP2, was isolated from MicroTom cell cultures, purified, and characterised. The enzyme was identified with 64% sequence coverage as the leprx21 gene product (suberization-associated anionic peroxidase 2-like) from *Solanum lycopersicum*, 334 amino acids long. Compared to other plant peroxidases, SAAP2 was more active at elevated temperatures, with the optimal temperature and pH at 90 °C and 5.0, respectively. Furthermore, the enzyme retained more than 80% of its maximal activity over the range of 70–80 °C and the presence of NaCl (1.0–4.5 M). It also exhibited broad pH versatility (65% relative activity over the pH range 2.0–7.0), acid-tolerance (80% residual activity after 22 h at pH 2.0–7.0), high thermostability (50% residual activity after 2 h at 80 °C) and proteolytic resistance. SAAP2 exhibited exceptional resistance under thermo-acidic conditions compared to the horseradish peroxidase benchmark, suggesting that it may find potential applications as a supplement or anti-pollution agent in the food industry.

## Introduction

Peroxidases (PODs) are ubiquitous enzymes belonging to the class of oxidoreductases, capable of catalysing a wide variety of oxygen-transfer reactions between hydrogen peroxide or other peroxides as electron acceptors and many kinds of substrates (xenobiotic, lignin and other phenolic compounds), resulting in oxygen (O_2_) liberation from H_2_O_2_^1^. The non-animal heme superfamily is one of the most studied groups of peroxidases, which includes enzymes from plant, fungi and bacteria, and it is further divided into three classes based on the origin, amino acid homology and metal-binding capability^2,3^. Within this group, enzymes from plant sources have been proposed for a wide range of industrial applications within areas such as health sciences, food industry and ecology. In particular, horseradish and soybean peroxidases have been employed as biotechnological tools in bio-sensing, as well as in the micro- and nano-sized structures in diagnostics, thanks to their stability compared to other characterized peroxidases^4^. However, compared to the horseradish peroxidase (HRP), the soybean peroxidases obtained from the seed coats exhibited a higher thermal and conformational stability due to their unique nature of their heme binding^5^. Other plant enzymes have also found promising potentialities in the environment protection, thanks to their abilities to degrade a broad range of toxic pollutants such as petroleum hydrocarbons, dioxins, industrial dye effluents, herbicides and pesticides^2,6^.

In this work a new tomato peroxidase LePrx21, named SAAP2, was isolated, purified and characterised from cell suspension cultures of tomato (*Solanum lycopersicum*) MicroTom cultivar. MicroTom is a miniature dwarf tomato plant which shares several unique features with Arabidopsis, such as a small size that enables it to grow at a high density in the laboratory and a short life cycle, making it a model system for Plant Biology and tomato functional genomics^7^. Moreover, plant cell culturing represents a valuable industrial technique to obtain bioactive compounds or extracts in higher amounts and with lower costs than those obtained by commonly used plant extraction processes^8^.

SAAP2, abundantly expressed by MicroTom cells grown as suspension cultures, revealed unusual extremophilic properties, such as optimal activity at 90 °C and high saline concentration up to 3.5 M, when examined under different physicochemical conditions and operational parameters. In addition, the enzyme exhibited exceptional resistance to high temperatures and acidic pH, which usually compromise the structural integrity and function of most proteins. These findings make SAAP2 a promising candidate for future studies on application fields, including therapeutics and environment bioremediation.

## Results and discussion

### Protein identification

**Figure 1:**
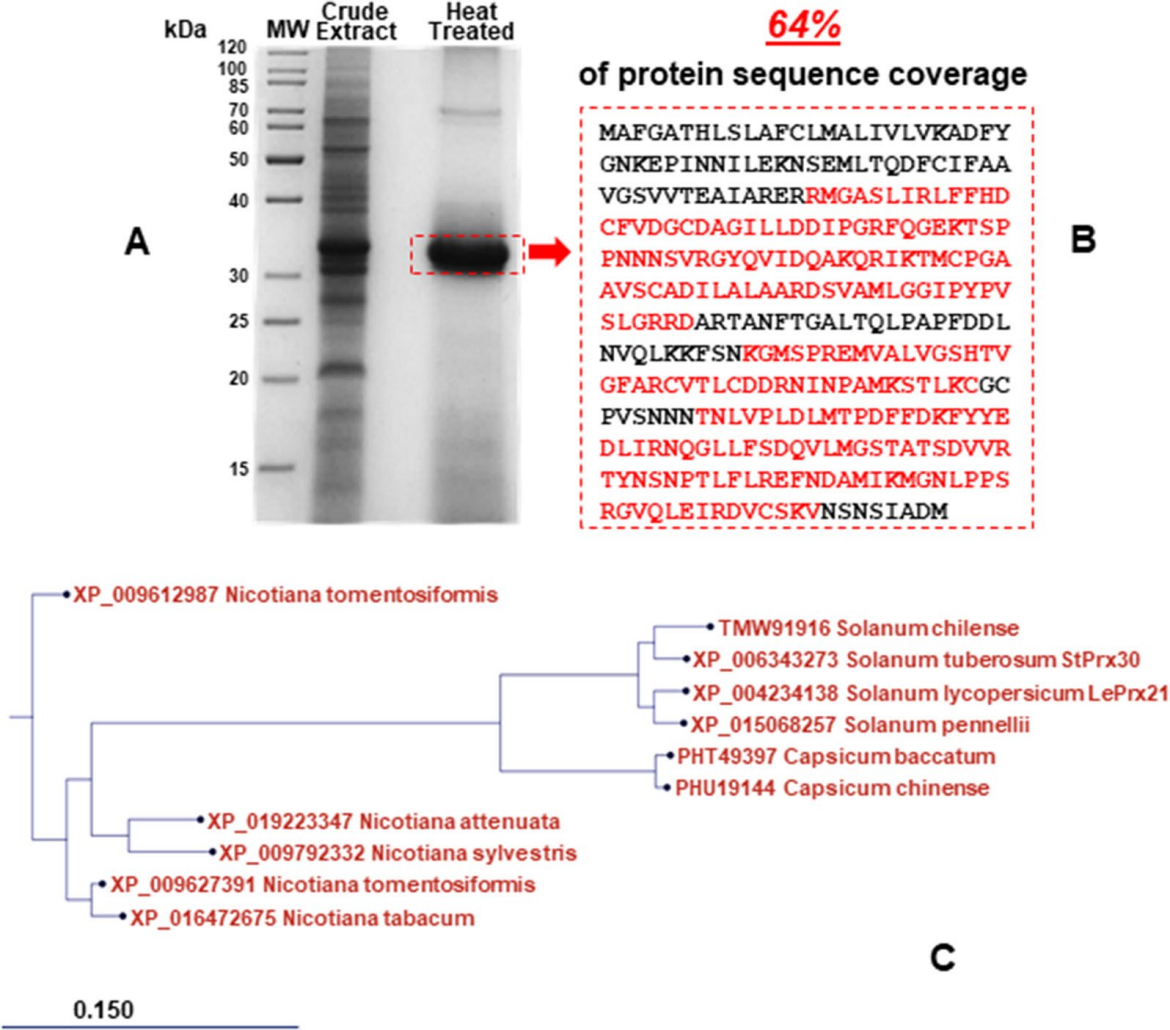
SDS-PAGE, sequence analysis and phylogenetic tree of SAAP2 from MicroTom cell cultures. (**A**) SDS-PAGE analysis of total protein extracts before and after thermal treatment at 80 °C for 30 min obtained from tomato cell lines. Total protein extracts (30 g) were electrophoresed on 12% SDS–polyacrylamide gel and detected by Coomassie blue staining. MW: molecular weight markers. (**B**) Amino acid sequence deduced from the peroxidase-coding gene (LePrx21). The peptides identified by LC–MS/MS are highlighted in red. (**C**) Phylogenetic tree from the amino acid sequences of LePrx21 and the highly homologous peroxidases. The scale bar indicates the genetic distance among the sequences used to construct the phylogram: a distance of 0.150 means that substitutions/changes for each amino acid between two taxa have a 15% probability.

During our previous study concerning the recombinant expression of archaeal Superoxide Dismutases in tomato cells^9^, an unexpected finding caught our attention and prompted us to investigate further. Indeed, when the non-transgenic protein extracts obtained from calli of MicroTom cotyledons were examined by SDS-PAGE, a protein band migrating with an apparent MW of 32.6 kDa appeared more intense and persistent despite treatment at 80 °C for 30 min, thus suggesting a strong thermotolerance as an exclusive feature of this protein in the crude mixture. To further explore this aspect, the protein identity was first determined by LC–MS/MS analysis, subjecting the aforementioned SDS-PAGE band to in-gel tryptic digestion (Fig. 1A). The Peptide Mass Fingerprint analysis of the tryptic peptides gave a significant match with peroxidase from *Solanum lycopersicum* and it was precisely recognized as the LePrx21 gene product, a suberization-associated anionic peroxidase 2-like (NCBI Reference Sequence: XP_004234138.1; UniProtKB accession A0A3Q7FFR5), which consisted of 334 amino acids and was implicated in the defence response to environmental stresses such as wounding, Mn excess^10^, pathogen attack or oxidative stress^11,12^. The total coverage of the protein sequence was 64% (Fig. 1B) and no significant contaminants were found in the 32.6 kDa-band excised from the gel, confirming a high degree of purity. In addition, the molecular mass determined by SDS-PAGE was in good agreement with that deduced from the LePrx21 amino acid sequence (36.5 kDa).

It is worth noting that despite the query “*Solanum lycopersicum* peroxidase” recalling 223 results on the UniProtKB database (https://www.uniprot.org/) and more than 450 articles on this topic having been published from 2012 to date (https://pubmed.ncbi.nlm.nih.gov/?term=), very few members of this huge gene family have been recognised and characterised in tomato. Specifically, in addition to the most extensively studied peroxidases such as the two enzymes identified in tomato fruits, LePrx75 (TAP2) and LePrx76 (TAP1)^13,14^ and the TPX1 detected in roots^15^, only four other components expressed in the fruits (LePrx09, LePrx17, LePrx35 and LePrxA) were recently characterised^16^.

The gene *leprx21* identified in this study (K4BE93_SOLLC, corresponding to genomic annotations LOC101265511 and Solyc03g006700.2.1) was found highly expressed in MicroTom cell cultures, as confirmed by the numerous hits from potato and tomato callus cDNA libraries obtained by blasting *leprx21* mRNA against the expressed sequence tags database (Supplementary Fig. 1)^17^. Moreover, as shown in the phylogenetic tree reported in Fig. 1C, LePrx21 was very close to the StPrx30 peroxidase isolated in potato (*Solanum tuberosum* M1A251_SOLTU).

To establish whether SAAP2, exhibiting uncommon ‘hyperthermophilic’ futures, was also expressed in other tomato cultivars, protein extracts from the Moneymaker cultivar cell cultures were analysed for peroxidase (POD) activity before and after heat treatment at 70 or 80 °C in comparison to those from MicroTom. As reported in Table 1 in Fig. 1, the specific activity estimated by the guaiacol assay strongly differed between the cultivars, showing a fourfold increase in the crude cell extract from MiroTom compared to that from Moneymaker. The peroxidase from MicroTom still retained its activity after the thermal treatments, remaining soluble with a remarkable improvement of the specific activity (3.4- and 5.4-fold increase at 70 or 80 °C, respectively, compared to the untreated sample), thus suggesting a thermal-stability of the enzyme expressed in this tomato cultivar.

**Figure 2:**
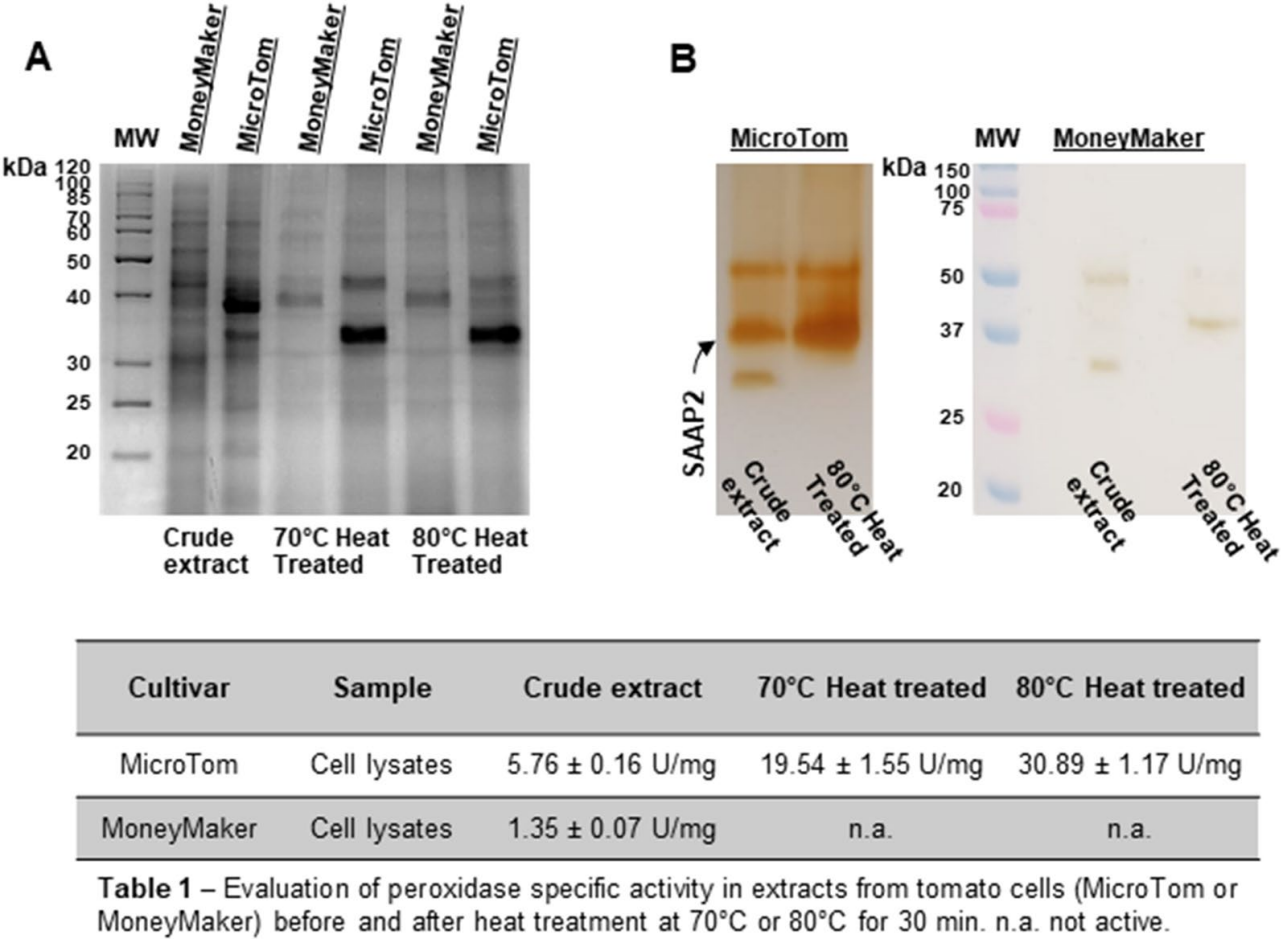
Electrophoretic analyses of total protein extracts obtained from cell lines of MicroTom and Moneymaker cultivars. (**A**) Total protein extracts (15 µg) before and after thermal treatment at 70 or 80 °C for 30 min were electrophoresed on 12% SDS–polyacrylamide gel and detected with Coomassie blue staining. MW: molecular weight markers. (**B**) Total protein extracts (15 µg) before and after thermal treatment at 80 °C for 30 min were electrophoresed on semi-native PAGE (12%). Following semi-native PAGE, protein bands were detected by in-gel POD activity staining using guaiacol as substrate. The results are representative of three independent experiments on three different protein extracts. Data were expressed as means ± standard deviation.

The samples obtained from both tomato cultivars were also evaluated before and after the thermal treatments by SDS-PAGE and semi-native PAGE followed by in-gel activity staining. The electrophoretic results confirmed those obtained previously with MicroTom, highlighting the presence of the intense protein band with an approximate molecular mass of 32.6 kDa, which was greatly evident in the heat-treated extracts (Fig. 2A) and resulted positive after in-gel POD activity staining, further confirming that SAAP2 was a peroxidase (Fig. 2B). However, it is worth noting that three distinct POD-activity bands were detected in tomato MicroTom extracts with an enrichment in activity of the 32.6 kDa-isoform after thermal treatment at 80 °C, which was less evident after incubation at 70 °C (data not shown). On the other hand, a thermostable peroxidase band activity (corresponding to a molecular mass of 39.0 kDa) was also detected in Moneymaker extracts only after heat treatment at 80 °C, although at very low intensity.

Indeed, the absence of an overexpressed peroxidase possibly corresponding to SAAP2 after heat treatment in Moneymaker could be due to either a different regulation of the *leprx21* gene in this tomato variety as compared to MicroTom or an inhibition of the enzymatic activity by post-translational modifications, presence of metabolites, or to a combination of these factors.

Moreover, a homology sequence analysis performed with BLAST on the genome of MicroTom and various Moneymaker SRA collections, using the cDNA of the SAAP2 reference gene as a query, revealed: (1) a complete sequence match between Moneymaker and the reference SAAP2 mRNAs (Supplementary Fig. 2A); (2) two amino acid substitutions in the MicroTom SAAP2 isoform at 43 (L43S) and 191 (N191D) positions (Supplementary Fig. 2B). However, these substitutions, although non-conservative and able to increase the polarity of the MicroTom isoform, should not affect the catalytic site of the enzyme, as evidenced by the analysis of the amino acid sequence alignment among the peroxidase isoforms (Supplementary Fig. 2C) and by a build-up 3D model of the MicroTom enzyme (Supplementary Fig. 2D). On the other hand, we cannot exclude that the observed mutations might play a role in modifying the flexibility of the structure, involved in electrostatic interactions.

### Extraction and purification of SAAP2 from MicroTom tomato cells

**Figure 3:**
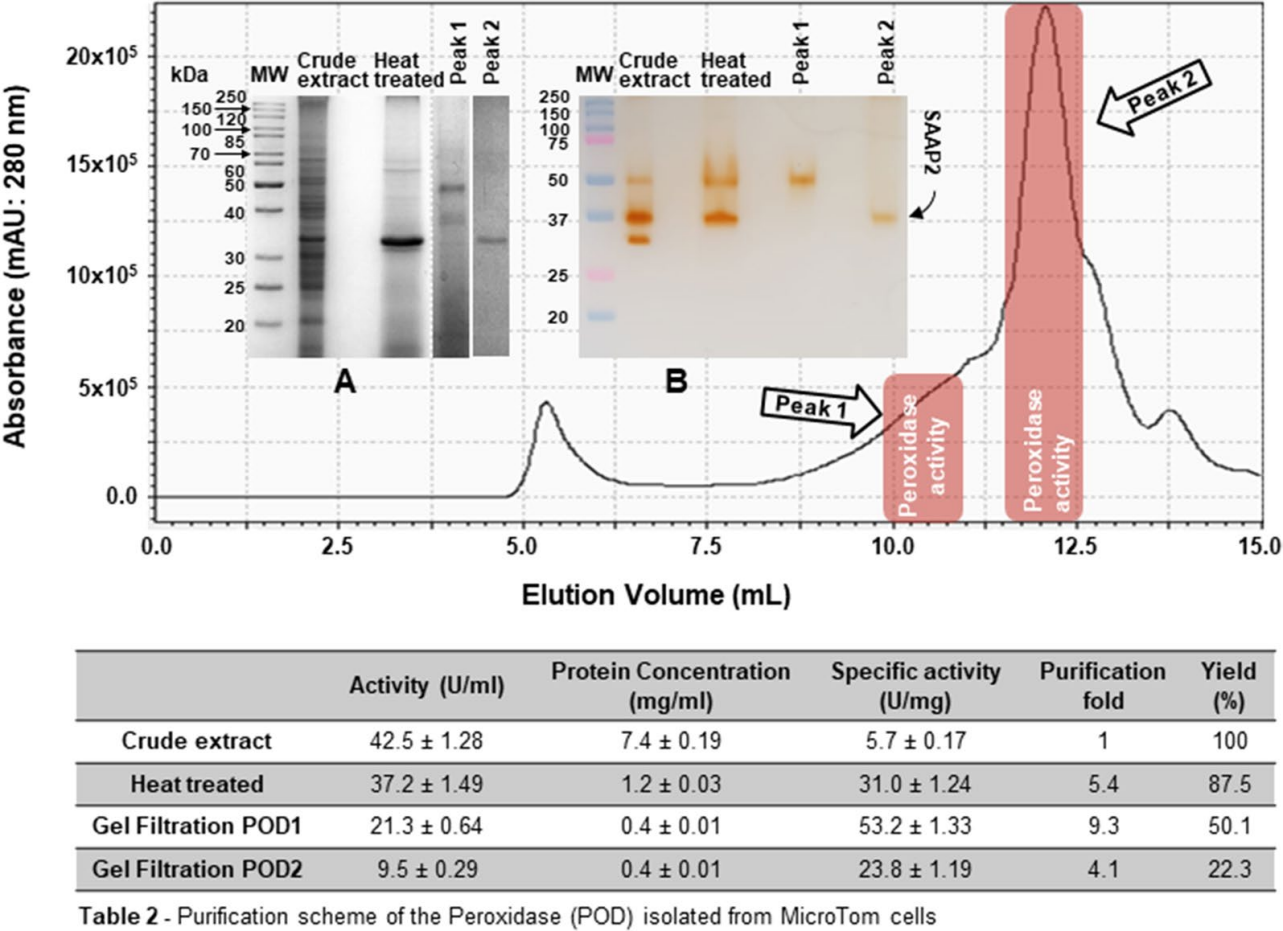
Gel filtration chromatography of crude extracts from MicroTom cells. Size-exclusion chromatography was performed on the YARRA™ SEC-4000 column in a 50 mM Tris–HCl buffer pH 7.5 containing 50 mM NaCl. The absorbance was measured at 280 nm. *Insert*: (**A**) SDS-PAGE (12%) and (**B**) semi-native PAGE (12%) analyses of MicroTom crude extracts before and after thermal treatment at 80 °C for 30 min and of PODs-fractions obtained after gel filtration chromatography. Following semi-native PAGE analysis, protein bands were detected by in-gel POD activity staining using guaiacol as substrate. *MW* molecular markers. The results are representative of three independent experiments on three different protein preparations. Data in Table 2 in Figure were expressed as means ± standard deviation.

To investigate on the biochemical properties of SSAP2 from MicroTom, a purification protocol was set up using total protein samples from suspension cell cultures^18^. A summary of the purification procedure is shown in Fig. 3. The lyophilised powder obtained as described in the “Methods” section, was dissolved in water at a concentration of 10% w/v and the resulting solution was heat treated, due to the high thermostability previously observed for the two peroxidases of 32.6 and 42.7 kDa from tomato cell cultures, with the 32.6 kDa enzyme being the most abundant as revealed by zymography and SDS-PAGE analyses (Fig. 2). The thermal treatment step helped to improve the peroxidase purification as most of the proteins from the crude extract were removed by precipitation, and the specific activity and purification-fold of the enzyme sample were recorded as 37.2 (U/mg) and 5.4-fold, respectively. The subsequent fractionation of the protein sample by gel filtration chromatography on a YARRA column resulted in the identification of two resolved POD-activity peaks (Fig. 3), characterized by molecular masses of 50 kDa (Peak1_POD1) and 30 kDa (Peak2_SAAP2). SDS-PAGE (Fig. 3A) and semi-native PAGE (Fig. 3B) analyses confirmed that the active protein fraction 2 corresponded to SAAP2, which appeared purified to homogeneity with a specific activity of 23.8 U/mg as reported in Table 2 in Fig. 3.

### Biochemical characterization of SAAP2

The biochemical characterization of the purified SAAP2 was assessed and compared to that of the HRP, which represented a good benchmark of a commercial plant-derived peroxidase with proved biotechnological applications. However, HRP may have some limitations regarding stability under certain conditions, hence the interest in finding novel peroxidases with similar applicability but higher stability.

**Figure 4:**
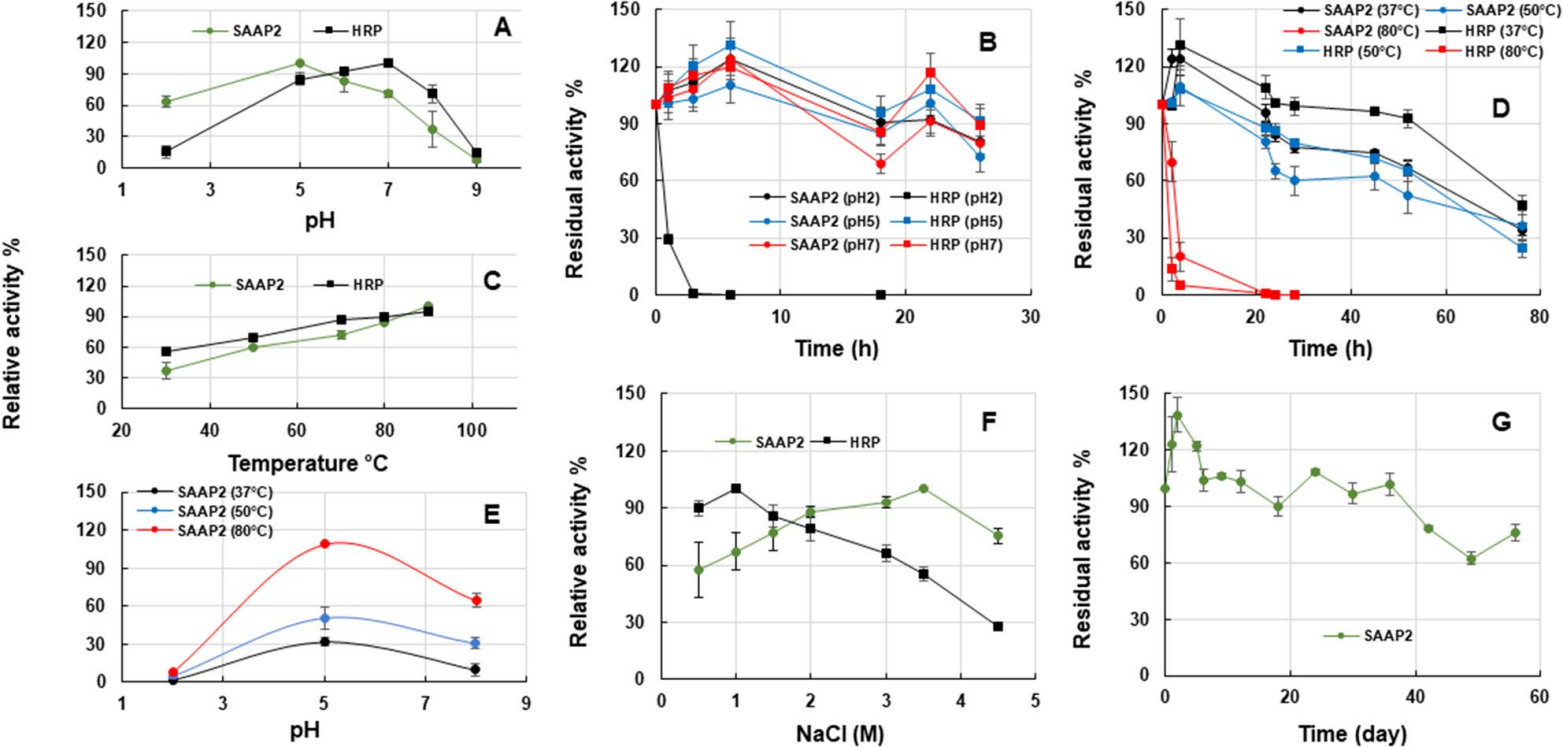
Biochemical characterization of SAAP2 in comparison with the commercially available horseradish peroxidase (HRP). (**A**) Effects of pH on the peroxidase activities. (**B**) Effects of pH on the stability of SAAP2 and HRP. (**C**) Effects of temperature on peroxidase activities. (**D**) Effects of temperature on the stability of SAAP2 and HRP. (**E**) Effects of pH and temperature on the SAAP2 activity. (**F**) Effects of ionic strength on peroxidase activities. **(G)** Storage stability at 4 °C of SAAP2. All experiments were performed in triplicate on three different protein preparations, with guaiacol as substrate. Relative activity was expressed as % of the highest activity that was set as 100%. The residual activity was expressed as % to the initial activity that was set as 100%.

It is widely known that pH is a critical factor in the determination of enzyme activity as it affects the ionisation state of amino acids and substrates^19^. Therefore, the effect of different pHs on the activity and stability of SAAP2 was determined in the range from 2.0 to 9.0, using guaiacol as substrate. As reported in Fig. 4A, the purified peroxidase was active over the pH range of 2.0–8.0, reaching its maximum at pH 5.0. The relative activity of SAAP2 resulted above 60% in the range 2–7.5, while it decreased at pH values higher than 8.0, after which the enzyme lost nearly 90% of its activity at pH 9.0, probably due to changes in the charge of the amino acid residues in the active site, resulting in the dissociation of the substrate or even changing its ionisation state. In alternative the activity loss may derive from protein denaturation or ionic shifts in the haem group^20^. For comparison, the commercial HRP exhibited maximum activity at pH 7, with a strong decrease observed at acidic and alkaline pHs, where it retained only 17% of its maximum activity.

To assess the pH-stability of the purified SAAP2 in comparison with that of HRP, both enzymes were incubated at three different pHs for 26 h. As shown in Fig. 4B, the purified peroxidase was stable at all the tested pH values, retaining more than 70% of its activity after 26 h of incubation. In the case of HRP, it showed complete inactivation after 3 h of incubation under extreme acidic conditions, probably due to the detachment of the heme group from the enzyme, while at pH 5.0 and 7.0 its behaviour was comparable to that of SAAP2. On the contrary, the high resistance at low pH suggested that SAAP2 could hold its heme group more tightly than many other plant PODs. Therefore, the effect of pH on the activity and stability of SAAP2 indicated that this enzyme could potentially suit different industrial applications that require acidic conditions, where HRP could not operate properly.

As far as the effect of temperature was concerned, the peroxidase activity was measured in the range of 30–90 °C. As depicted in Fig. 4C, SAAP2 activity increased quickly when the temperature rose from 30 to 90 °C, reaching its maximum at 90 °C, thus suggesting that no thermal denaturation phenomenon occurred at the enzyme expenses.

A similar trend and an optimum temperature of 90 °C were observed for HRP. Moreover, the thermal stability was determined by incubating the peroxidases for 76 h at different temperatures. As shown in Fig. 4D, SAAP2 was stable at 37 °C and 50 °C. After 22 h incubation at 37 °C, almost 100% activity was maintained, and 84% at 50 °C, while more than 40% of its original activity was retained after 76 h incubation at both temperatures. These results are comparable to those obtained in the same temperature ranges with HRP. On the contrary, 50% of activity was still retained after 2 h incubation at 80 °C for SAAP2, while a sharp and considerable loss of activity was detected for HRP. A complete inactivation of SAAP2 at 80 °C occurred only with increasing incubation times up to 22 h. Therefore, these results suggested that only minor differences were observed in the thermal response between SAAP2 and HRP. To better investigate the simultaneous effect of temperature and pH on SAAP2 activity, assays were conducted in the ranges of pH 2.0–8.0 and temperature 37–80 °C. Data reported in Fig. 4E demonstrated that the enzyme exhibited a scarce relative activity at pH 2.0 and in the range 37–80 °C, suggesting that the combined effect of temperature and acid pH influenced the activity of SAAP2. It can be assumed that SAAP2 may work properly in applications requiring weak acidic or alkaline conditions and operative temperatures from 37 to 80 °C, for example the removal of organic pollutants in wastewater.

To study the effect of ionic strength on peroxidase activity, different concentrations of NaCl (0.5–4.5 M) were set up in the reaction mixture. As shown in Fig. 4F, the SAAP2 activity increased linearly with the salt concentrations, until achieving maximum at 3.5 M and retaining 80% activity with a 4.5 M concentration, indicating that the salt addition did not affect the activity of POD. Conversely, the activity of HRP was highest at the saline concentration of 1.0 M, varied slightly in the range from 0.2 to 2.0 M, and gradually decreased over 2.0 M, reaching 30% at 4.5 M. Finally, the influence of 56 days of storage at 4 °C on SAAP2 activity was investigated. As reported in Fig. 4G, the enzyme was stable under the applied storage conditions, with almost full activity remaining after 56 days of storage, thus broadening the spectrum of its potential industrial applications with relevant economic impact.

**Figure 5:**
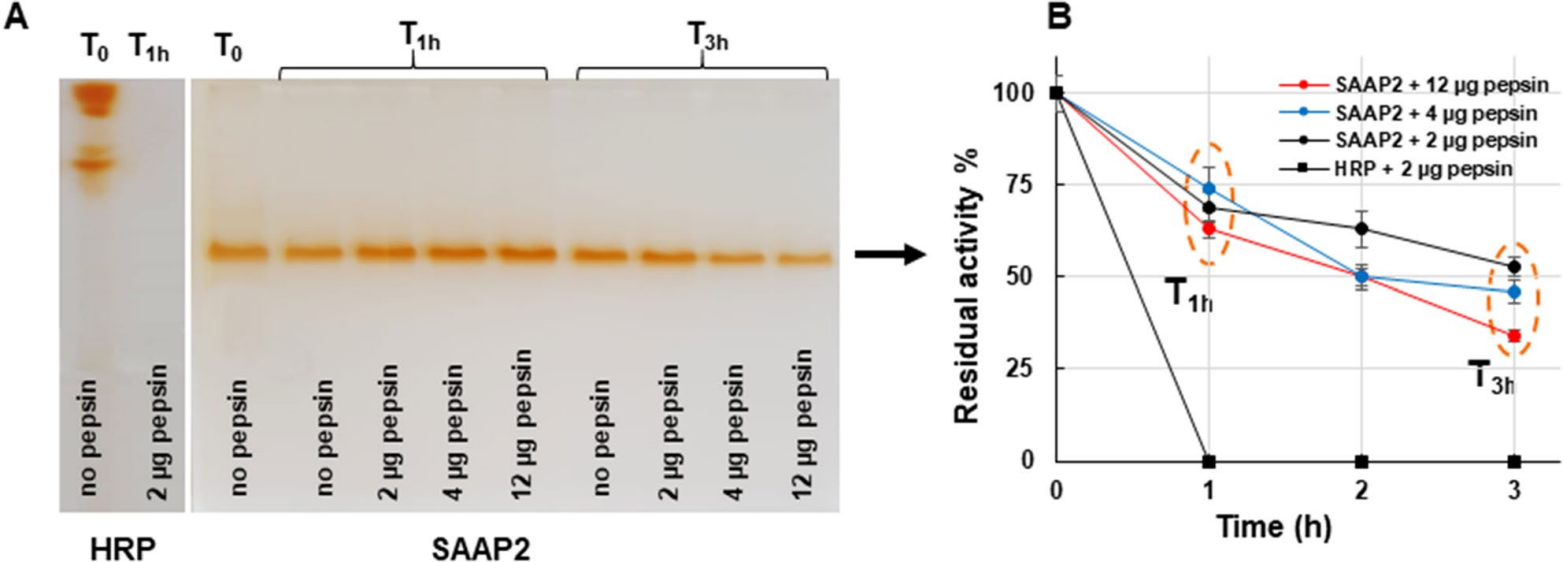
Proteolytic resistance of SAAP2. (**A**) Semi-native PAGE analysis of the reaction mixtures (15 µg) obtained by incubating the purified SAAP2 (70 µg) without (T0) or with different concentrations of pepsin (2 µg, 4 µg and 12 µg) up to 3 h at 37 °C, all the sample analysed on the gel were incubated at pH 2.0. Following semi-native PAGE, protein bands were detected by in-gel POD activity staining using guaiacol as substrate. (**B**) Spectrophotometric analysis of purified SAAP2 (70 µg) incubated up to 3 h at 37 °C, all samples were incubated at pH 2.0 and treated with different concentrations of pepsin (2 µg, 4 µg and 12 µg). The oxidation of guaiacol was followed by observing the increase in absorbance at 470 nm. The benchmark HRP (70 µg) incubated up to 3 h at 37 °C and pH 2.0 with pepsin (2 µg) was used as control. Residual activity was calculated relative to the initial activity (T0) that was set as 100%. The results are representative of three independent experiments on three different protein preparations.

To further investigate on the biochemical properties of SAAP2, we conducted a study to evaluate its resistance to acidic proteolysis by pepsin, the main enzyme involved in gastric digestion. The purified SAAP2 was incubated for 3 h at pH 2.0 and 37 °C in the presence or absence of different amounts of pepsin and then analysed by spectrophotometric assay and semi-native PAGE, followed by in gel-POD activity. Results with zymography clearly demonstrated that SAAP2 preserved its activity in the presence of all the pepsin concentrations used after 1 h of incubation, while about a 50% reduction of POD band intensity was observed at 12 and 4 µg of pepsin (Fig. 5A,B). Conversely, the HRP benchmark was totally inactivated already after 1 h of incubation with the lowest pepsin concentration tested, thus confirming the stability of SAAP2 to acid pHs and suggesting a noticeable and unusual resistance of this peroxidase to protease degradation. In addition, the spectrophotometric measurements of the samples were in good agreement with those observed with the electrophoretic method, validating the general susceptibility to proteolytic degradation of mesophilic proteins and underlying the biotechnological potential of SAAP2**.**

### Screening of peroxidase from various vegetable sources

The widespread use of peroxidase in biotechnology, medicine and industry has encouraged scientists and industries to find novel enzymes from this family, keeping in mind their importance. Due to the difficulty of synthesis and costs of many important industrial enzymes, an increasing number of researchers are investigating new natural sources^21,22^, such as plants^23^.

**Figure 6:**
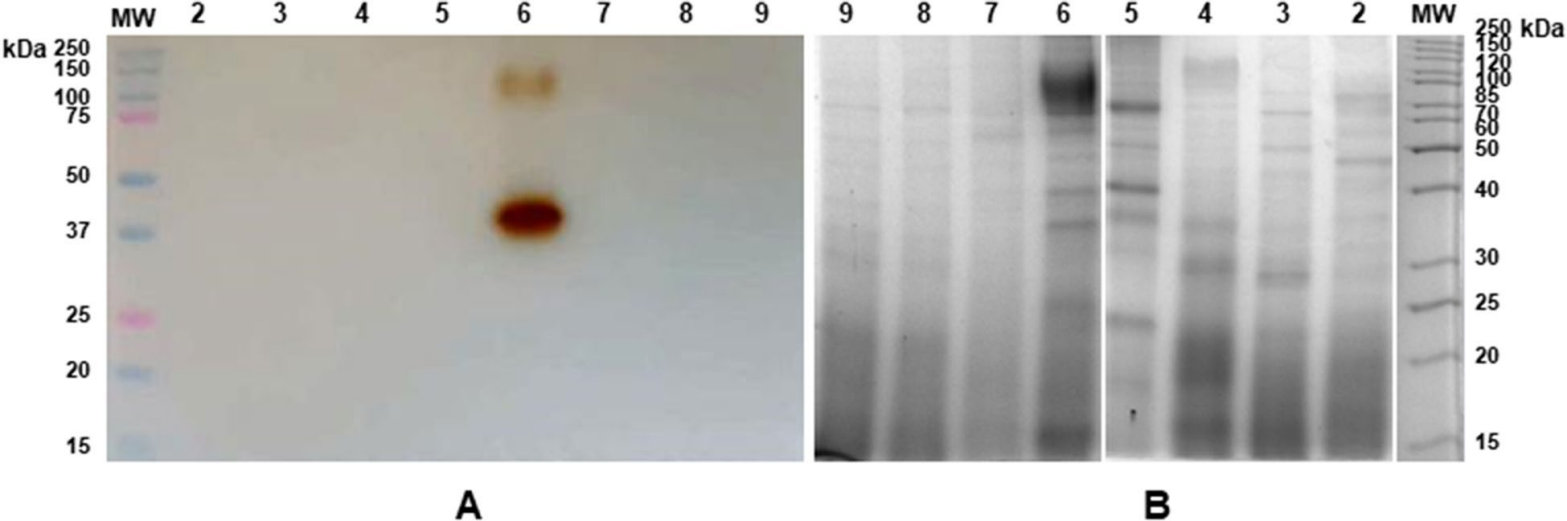
Electrophoretic analysis of heat-treated crude extracts obtained from different plant samples. Total protein extracts (15 µg) obtained after thermal treatment at 80 °C for 30 min from cell cultures of different plants were electrophoresed. (**A**) semi-native PAGE (12%) analysis followed by in-gel POD activity staining using guaiacol as substrate. Lane 1: molecular weight markers; Lane 2: *Daphne odora*; Lane 3: *Rubus idaeus*; Lane 4: *Psilanthus bengalensis*; Lane 5: *Pelargonium capitatum*; Lane 6: *Solanun melanogena*; Lane 7: *Cirsium eriophorum*; Lane 8: *Ficus carica* grown in the light; Lane 9: *Ficus carica* grown in the dark. (**B**) SDS-PAGE (12%) analysis followed by Coomassie blue staining. The lane numbers correspond to the same samples reported in (**A**). The results are representative of three independent experiments on three different protein extracts.

In order to investigate whether peroxidases showing ‘hyperthermophilic’ properties similar to those exhibited by SAAP2 were present in other plant species as well, a preliminary screening and comparison of peroxidase activity was performed on the extracts of cell suspension cultures obtained from the following plant species: *Daphne odora, Rubus idaeus, Psilanthus bengalensis, Pelargonium capitatum, Solanum melongena, Cirsium eriophorum, Ficus carica.* Specifically, the crude extracts were subjected to heat treatment at 80 °C and then analysed by semi-native PAGE followed by in gel-activity staining (Fig. 6A). Results clearly indicated that among all the samples investigated, only the extract from *Solanum melongena* exhibited a strong peroxidase activity that resisted to a pre-treatment at 80 °C. However, all the above cell extracts analysed by SDS-PAGE evidenced the presence of heat-resistant protein bands that probably did not include peroxidase isoforms except for *Solanum melongena* samples (Fig. 6B), thus confirming the results reported in Fig. 6A.

## Conclusion

Due to the ability of catalysing a wide range of biochemical reactions, peroxidases are considered valuable enzymes in the industrial field. In this context, the discovery of PODs with peculiar characteristics is important to provide new sources for future applications. In this study, a novel POD named SAAP2, specifically produced in MicroTom cultivar cell cultures and not yet described, was identified, purified and biochemically characterised in comparison with the commercially available benchmark HRP, elucidating its unusual biochemical properties of high thermal stability, wide pH resistance, high salt tolerance, and long shelf-life, which are quite uncommon properties associated to plant peroxidases.

The most thermostable peroxidases so far characterized are those belonging to soybean: they possess a high melting temperature of 90.5 °C at pH 8.0, probably due to an intrinsic capacity to holds onto its heme much more tightly than other similar enzymes.

Similarly, a peroxidase from *Ficus sycomorus* latex showed high thermal stability with no loss of enzyme activity recorded up to 60 °C, although the pH range of activity was restricted from 5.5 to 7.0 and no salt tolerance was described^24^. Another peroxidase isolated from garlic bulb (*Allium sativum* L.) extract showed to be stable over a pH range of 3.5–11.0 but lost 50% of its activity at 50 °C^25^. As additional example, a soluble and thermostable peroxidase from *Citrus medica* was active in the range of pH 5.0–8.0 and temperature 30–80 °C, however was readily inactivated at 60–75 °C^26^.

In conclusion, although SAAP2 showed similar thermo-stability properties as other known plant peroxidases, the features of acid pH resistance, high salt concentration tolerance and stability over-time appear to be unique and not yet reported for other plant peroxidases. Taken these properties together with the advantage of using plant cells as a bio-factory, this enzyme may find interesting applications including human health and environment.

## Methods

### Production of tomato cotyledons

The procedure of obtaining the calluses of *Solanum lycopersicum* starting from sterilized seeds was carried out essentially according as previously described^18^.

### Production of the tomato and vegetables cell extracts

To initiate plant cell cultures the same protocol adopted in Palmieri et al. was used^18^. The resulting lysate was centrifuged and the supernatant was collected and lyophilized^18^. The lyophilized powder was then dissolved in water at a concentration of 10% w/v and the protein concentration was measured by the Bradford assay^27^, using BSA as standard. For the thermal treatment, the extracts were incubated for 30 min at 80 °C and then the mixtures were centrifuged at 10,000×*g* to separate the precipitated proteins and obtain the soluble components. All the extracts obtained from the calli of the other vegetable species used in this study (*Daphne odora*, *Rubus idaeus*, *Psilanthus bengalensis*, *Pelargonium capitatum*, *Solanun melanogena*, *Cirsium eriophorum*, *Ficus carica* grown in the light and in the dark) were prepared as described for those of tomato cultivars.

### SDS-PAGE and semi-native PAGE analyses

Thirty micrograms of the total protein extracts were loaded onto 12% SDS-PAGE gel and detected with Coomassie blue staining.

For semi-native PAGE analysis followed by in-gel POD activity, an equal amount (15 µg) of each sample was separated by electrophoresis on 12% SDS-PAGE, but the sample was prepared like for native gel (i.e. without denaturing conditions and without boiling at 100 °C). Then, the gel was washed twice in Triton 1% for 15 min and then soaked at room temperature in the staining solution containing 25 mM sodium phosphate buffer pH 7.0, 5 mM of guaiacol and 20 mM H2O2. The POD bands were visualised as reddish bands in a clear background.

Cropped images of SDS-PAGE or semi-native PAGE gels were reported in the paper for a better editing of the figures.

### HPLC-MS/MS analysis

SAAP2 protein band stained with Coomassie Brilliant Blue G250 was cut from SDS-PAGE (12%) and subjected to in situ digestion as previously reported^28^. The obtained peptide mixtures were collected, vacuum-dried, and then resuspended in 0.2% formic acid for LC–MS/MS analyses which were carried out onto an LTQ Orbitrap XL (ThermoScientific, Waltham, MA, USA) coupled with a nanoLC system (nanoEasy II). Peptide mixtures were separated onto a C18 capillary column (200 mm, 75 µm ID, 5 µm, 120 Å, Nanoseparation, NL) by using a linear gradient of eluent B (0.2% formic acid in 95% acetonitrile LC–MS Grade) from 5 to 95% in 87 min at a flow rate of 250 nL/min. The mass spectrometry analyses were performed using the data-dependent acquisition (DDA) mode: from each MS scan in the range from 400 to 1800 m/z, the ten most abundant ions were selected and fragmented in collision-induced dissociation (CID) conditions, applying a dynamic exclusion window of 40 s. Raw data obtained from nanoLC-MS/MS were analysed with MaxQuant 1.5.2 integrated with the Andromeda search engine, generating the peak lists which were used for tandem mass spectrometry (MS/MS) ion search in the Mascot Server for protein identification.

### Phylogenetic analysis

Phylogenetic tree was calculated with Phylogeny 1.3 on the CLC Main Workbench 23.0.3 (QIAGEN Aarhus, Denmark), by Maximum Likelihood (ML) with Neighbor Joining construction method; Jukes Cantor nucleotide substitution model; WAG protein substitution model; transition/transversion ratio = 2,0; no rate variation; number of substitution rate categories = 4; gamma distribution parameter = 1,0; by estimating substitution rate parameter(s) and topology; by performing bootstrap analysis with 100 replicates.

### Sequence analysis of SAAP2 isoforms

BLAST analyses (Basic Local Alignment Search Tool; https://blast.ncbi.nlm.nih.gov/blast/Blast.cgi) were performed scanning chromosome 3 whole genome shotgun of cultivar MicroTom (accessions: CM022784.1 and JAAXDC010000007.1) and three Sequence Read Archive (SRA) collections of cultivar Moneymaker (accessions: SRR18779969, SRR18779970 and SRR18779971) using the cDNA of SAAP2 reference gene (cultivar Heinz 1706) as a query (XM_004234090 SOLLC mRNA_LOC101265511). The amino acid sequences of SAAP2 isoforms of the three tomato cultivars were aligned to HRP by ClustalO on CLC Main Workbench 23.0.3 (QIAGEN Aarhus, Denmark). Finally, a model of MicroTom SAAP2 was create on the same software package.

### Peroxidase assay and protein determination

Peroxidase activity was determined using guaiacol as reducing substrate in a reaction mixture containing 25 mM sodium phosphate buffer pH 7.0, 5 mM guaiacol and 20 mM H2O2. The oxidation of guaiacol to tetraguaiacol was followed by observing the increase in absorbance (*A*) at 470 nm (ε470 nm = 26.6 mmol/L/cm) and 30 °C, using a Jasco UV/Vis spectrophotometer. The blank sample contained all the reagents, except the enzyme. The enzyme unit was defined as the absorbance change at 470 nm/min produced by a given amount of enzyme, under the above assay conditions. The protein concentration was determined by the Bradford method^27^, using bovine serum albumin (BSA) as a standard. One unit (U) of enzyme activity was defined as the amount of enzyme that catalyses the oxidation of 1 µmol of guaiacol per min at 30 °C. Specific activity was defined as the amount of substrate that was oxidised per min by 1 mg enzyme.

### Biochemical characterization of SAAP2

The influence of pH on the enzymatic activity of SAAP2 was determined by measuring the formation of oxidised product of guaiacol at 30 °C in buffers at various pH values ranging from pH 2.0 to 9.0. The buffers used were at a concentration of 25 mM as the following: glycine–HCl (pH 2.0); sodium acetate (pH 5.0 and pH 6.0); sodium phosphate (pH 7.0); Tris–HCl (pH 8.0and 9.0). The effect of pH on SAAP2 stability was assayed by pre-incubating the purified enzyme in the above buffers at 37 °C for different times (0–26 h). Then, the residual activity was measured under the standard conditions, as described in the paragraph of “Peroxidase assay and protein determination” section. The optimal temperature was determined by measuring the enzyme activity under different temperatures from 30 to 90 °C. The temperature stability was determined by incubating the enzyme solution at various temperatures (37, 50 and 80 °C) for different times (0–76 h). After the incubation, the residual activity was followed as previously described. The effect of ionic strength on the SAAP2 activity was evaluated by adding different concentrations of NaCl (0.5–4.5 M) into the standard reaction mixture. The simultaneous effect of pH and temperature on enzyme activity was determined at a pH of 2.0, 5.0 and 8.0 at three temperatures (37, 50 and 80 °C). After the incubation, the residual activity was followed as previously described. For storage stability determination, the purified enzyme was first stored at 4 °C for 56 days in water at a concentration of 1 mg/mL and then the residual activity was measured. All the analyses were performed in comparison with the commercially available HRP that was used as a reference peroxidase. The relative activity was expressed as a percentage of the corresponding maximal activities of the enzyme under the standard assay conditions. The residual activity was expressed as a percentage of the corresponding initial activity that was set as 100%. All experiments were performed in triplicate on three different protein preparations.

The proteolytic stability of SAAP2 was assessed by incubating 70 µg of protein with the commercially available porcine pepsin (12 µg, 4 µg and 2 µg) at 37 °C up to 3 h. Then, the reaction mixtures (15 µg) were analysed by semi-native PAGE followed by in gel-POD activity and by spectrophotometric guaiacol assay. The same procedure was followed using HRP benchmark (70 µg) as reference protein, and the reaction mixtures were analysed by spectrophotometric method.

### Gel filtration chromatography

The size exclusion chromatography was performed on a YARRA™ SEC-4000 column (Pharmacia Biotech, Milan, Italy) pre-equilibrated with 50 mM Tris–HCl buffer (pH 7.5) containing 50 mM NaCl. Standard protein markers (BioRad code 151–1901) were used to calibrate the gel filtration column.

### Statistical analysis

Experiments were carried out in triplicate, on three different protein preparations. The results were expressed as means ± standard deviations (SD).

### Compliance declaration with international guidelines of research on plants

Authors declare that all plant experiment were conducted in accordance with relevant institutional, national, and international guidelines and legislation.

### Supplementary Information

Supplementary Figure 1.

Supplementary Figure 2.

Supplementary Figure 3.

Supplementary Figure 4.

Supplementary Information.

## Data Availability

Data is available in the Supplementary Files with the manuscript. The datasets used and/or analysed during the current study is available from the corresponding author on reasonable request.
