# Supplementary material for: A new versatile peroxidase with extremophilic traits over-produced in MicroTom cell cultures
Source: Sci Rep. 2023 Sep 15;13:15338. doi: 10.1038/s41598-023-42597-x (PMC10504257; doi:10.1038/s41598-023-42597-x)
Supplement: Supplementary file 1 — Supplementary Figure 1. [file 41598_2023_42597_MOESM1_ESM.pdf]

Suppl. Fig. A. Blastn analysis of various Moneymaker SRA collections using the cDNA of SAAP2 reference gene as a query. In green the direct sequences while in red those in the reverse direction. The amino acid translations are indicated.

LOC101265511

20 40 60

XM_004234090 SOLLC mRNA_LOC101265511	ATGGCTTTTGGTGCAACTCATTTGAGCTTGGCATTTTGCCTTATGGCTCTAATTGTTCTTGTAAAAGCAG
Translation ORF/CDS	M A F G A T H L S L A F C L M A L I V L V K A
Consensus	ATGGCTTTTGGTGCAACTCATTTGAGCTTGGCATTTTGCCTTATGGCTCTAATTGTTCTTGTAAAAGCAG
Translation ORF/CDS	M A F G A T H L S L A F C L M A L I V L V K A
SRA:SRR18779971.23685049.2	ATGGCTTTTGGTGCAACTCATTTGAGCTTGGCATTTTGCCTTATGGCTCTAATTGTTCTTGTAAAAGCAG
Translation ORF/CDS	M A F G A T H L S L A F C L M A L I V L V K A
SRA:SRR18779971.23685049.1	GCTTGGCATTTTGCCTTATGGCTCTAATTGTTCTTGTAAAAGCAG
Translation ORF/CDS	XX L A F C L M A L I V L V K A
SRA:SRR18779969.8691412.2	ATGGCTCTAATTGTTCTTGTAAAAGCAG
Translation ORF/CDS	M A L I V L V K A
SRA:SRR18779971.4811095.2	
Translation ORF/CDS	
SRA:SRR18779971.11429211.2	
Translation ORF/CDS	
SRA:SRR18779971.22392603.1	
Translation ORF/CDS	
SRA:SRR18779971.23396592.1	
Translation ORF/CDS	
SRA:SRR18779971.22392603.2	
Translation ORF/CDS	
SRA:SRR18779971.21820934.1	
Translation ORF/CDS	
SRA:SRR18779971.21621599.2	
Translation ORF/CDS	
SRA:SRR18779971.23641517.2	
Translation ORF/CDS	
SRA:SRR18779971.21621599.1	
Translation ORF/CDS	
SRA:SRR18779971.21820934.2	
Translation ORF/CDS	
SRA:SRR18779971.21291262.1	
Translation ORF/CDS	
SRA:SRR18779971.23641517.1	
Translation ORF/CDS	
SRA:SRR18779971.21291262.2	
Translation ORF/CDS	
SRA:SRR18779970.2541555.2	
Translation ORF/CDS	
SRA:SRR18779971.8085033.2	
Translation ORF/CDS	

LOC101265511

80 100 120 140

XM_004234090 SOLLC mRNA_LOC101265511	ATTTTTATGGTAACAAAGAGCCAATTAACAATATTTTGGAGAAAAATTCAGAAATGTTAACTCAAGATTT
Translation ORF/CDS	D F Y G N K E P I N N I L E K N S E M L T Q D F
Consensus	ATTTTTATGGTAACAAAGAGCCAATTAACAATATTTTGGAGAAAAATTCAGAAATGTTAACTCAAGATTT
Translation ORF/CDS	D F Y G N K E P I N N I L E K N S E M L T Q D F
SRA:SRR18779971.23685049.2	ATTTTTATGGTAACAAAGAGCCAATTAACAATATT
Translation ORF/CDS	D F Y G N K E P I N N I
SRA:SRR18779971.23685049.1	ATTTTTATGGTAACAAAGAGCCAATTAACAATATTTTGGAGAAAAATTCAGAAATGTTAACTCAAGATTT
Translation ORF/CDS	D F Y G N K E P I N N I L E K N S E M L T Q D F
SRA:SRR18779969.8691412.2	ATTTTTATGGTAACAAAGAGCCAATTAACAATATTTTGGAGAAAAATTCAGAAATGTTAACTCAAGATTT
Translation ORF/CDS	D F Y G N K E P I N N I L E K N S E M L T Q D F
SRA:SRR18779971.4811095.2	
Translation ORF/CDS	
SRA:SRR18779971.11429211.2	
Translation ORF/CDS	
SRA:SRR18779971.22392603.1	
Translation ORF/CDS	
SRA:SRR18779971.23396592.1	
Translation ORF/CDS	
SRA:SRR18779971.22392603.2	
Translation ORF/CDS	
SRA:SRR18779971.21820934.1	
Translation ORF/CDS	
SRA:SRR18779971.21621599.2	
Translation ORF/CDS	
SRA:SRR18779971.23641517.2	
Translation ORF/CDS	
SRA:SRR18779971.21621599.1	
Translation ORF/CDS	
SRA:SRR18779971.21820934.2	
Translation ORF/CDS	
SRA:SRR18779971.21291262.1	
Translation ORF/CDS	
SRA:SRR18779971.23641517.1	
Translation ORF/CDS	
SRA:SRR18779971.21291262.2	
Translation ORF/CDS	
SRA:SRR18779970.2541555.2	
Translation ORF/CDS	
SRA:SRR18779971.8085033.2	
Translation ORF/CDS	

LOC101265511

	160 · 180 · 200
XM_004234090 SOLLC mRNA_LOC101265511	TTGTATTTTCGCGGCGGTTGGATCTGTTGTGACGGAAGCTATTGCAAGGGAGAGGCGAATGGGAGCGTCT
Translation ORF/CDS	C I F A A V G S V V T E A I A R E R R M G A S
Consensus	TTGTATTTTCGCGGCGGTTGGATCTGTTGTGACGGAAGCTATTGCAAGGGAGAGGCGAATGGGAGCGTCT
Translation ORF/CDS	C I F A A V G S V V T E A I A R E R R M G A S
SRA:SRR18779971.23685049.2	
Translation ORF/CDS	
SRA:SRR18779971.23685049.1	TTGTATTTTCGCGGCGGTTGGATCTGTTGTGACGG
Translation ORF/CDS	C I F A A V G S V V T X
SRA:SRR18779969.8691412.2	TTGTATTTTCGCGGCGGTTGGATCTGTTGTGACGGAAGCTATTGCAAGGGAG
Translation ORF/CDS	C I F A A V G S V V T E A I A R E
SRA:SRR18779971.4811095.2	GCAAGGGAGAGGCGAATGGGAGCGTCT
Translation ORF/CDS	A R E R R M G A S
SRA:SRR18779971.11429211.2	CGAATGGGAGCGTCT
Translation ORF/CDS	R M G A S
SRA:SRR18779971.22392603.1	GGGAGCGTCT
Translation ORF/CDS	X G A S
SRA:SRR18779971.23396592.1	
Translation ORF/CDS	
SRA:SRR18779971.22392603.2	
Translation ORF/CDS	
SRA:SRR18779971.21820934.1	
Translation ORF/CDS	
SRA:SRR18779971.21621599.2	
Translation ORF/CDS	
SRA:SRR18779971.23641517.2	
Translation ORF/CDS	
SRA:SRR18779971.21621599.1	
Translation ORF/CDS	
SRA:SRR18779971.21820934.2	
Translation ORF/CDS	
SRA:SRR18779971.21291262.1	
Translation ORF/CDS	
SRA:SRR18779971.23641517.1	
Translation ORF/CDS	
SRA:SRR18779971.21291262.2	
Translation ORF/CDS	
SRA:SRR18779970.2541555.2	
Translation ORF/CDS	
SRA:SRR18779971.8085033.2	
Translation ORF/CDS	

LOC101265511

220 240 260 280

```
XM_004234090 SOLLC mRNA_LOC101265511 CTCATTCGCCTCTTCTTCCACGACTGCTTTGTCGATGGATGTGATGCTGGAATTCTTCTAGATGATATTC
                     Translation ORF/CDS  L  I  R  L  F  F  H  D  C  F  V  D  G  C  D  A  G  I  L  L  D  D  I

                            Consensus CTCATTCGCCTCTTCTTCCACGACTGCTTTGTCGATGGATGTGATGCTGGAATTCTTCTAGATGATATTC
                     Translation ORF/CDS  L  I  R  L  F  F  H  D  C  F  V  D  G  C  D  A  G  I  L  L  D  D  I

           SRA:SRR18779971.23685049.2
                     Translation ORF/CDS

           SRA:SRR18779971.23685049.1
                     Translation ORF/CDS

            SRA:SRR18779969.8691412.2
                     Translation ORF/CDS

            SRA:SRR18779971.4811095.2 CTCATTCGCCTCTTCTTCCACGACTGCTTTGTCGATGGATGTGATGCTGGAATTCTTCTAGATGATATTC
                     Translation ORF/CDS  L  I  R  L  F  F  H  D  C  F  V  D  G  C  D  A  G  I  L  L  D  D  I

           SRA:SRR18779971.11429211.2 CTCATTCGCCTCTTCTTCCACGACTGCTTTGTCGATGGATGTGATGCTGGAATTCTTCTAGATGATATTC
                     Translation ORF/CDS  L  I  R  L  F  F  H  D  C  F  V  D  G  C  D  A  G  I  L  L  D  D  I

           SRA:SRR18779971.22392603.1 CTCATTCGCCTCTTCTTCCACGACTGCTTTGTCGATGGATGTGATGCTGGAATTCTTCTAGATGATATTC
                     Translation ORF/CDS  L  I  R  L  F  F  H  D  C  F  V  D  G  C  D  A  G  I  L  L  D  D  I

           SRA:SRR18779971.23396592.1                                                               ATGATATTC
                     Translation ORF/CDS                                                               XX D  I

           SRA:SRR18779971.22392603.2
                     Translation ORF/CDS

           SRA:SRR18779971.21820934.1
                     Translation ORF/CDS

           SRA:SRR18779971.21621599.2
                     Translation ORF/CDS

           SRA:SRR18779971.23641517.2
                     Translation ORF/CDS

           SRA:SRR18779971.21621599.1
                     Translation ORF/CDS

           SRA:SRR18779971.21820934.2
                     Translation ORF/CDS

           SRA:SRR18779971.21291262.1
                     Translation ORF/CDS

           SRA:SRR18779971.23641517.1
                     Translation ORF/CDS

           SRA:SRR18779971.21291262.2
                     Translation ORF/CDS

            SRA:SRR18779970.2541555.2
                     Translation ORF/CDS

            SRA:SRR18779971.8085033.2
                     Translation ORF/CDS
```

	LOC101265511
	300 320 340
XM_004234090 SOLLC mRNA_LOC101265511	CTGGAAGGTTCCAAGGAGAAAAAACATCACCACCAAATAATAATTCAGTGAGAGGTTATCAAGTAATTGA
Translation ORF/CDS	P G R F Q G E K T S P P N N N S V R G Y Q V I D
Consensus	CTGGAAGGTTCCAAGGAGAAAAAACATCACCACCAAATAATAATTCAGTGAGAGGTTATCAAGTAATTGA
Translation ORF/CDS	P G R F Q G E K T S P P N N N S V R G Y Q V I D
SRA:SRR18779971.23685049.2	
Translation ORF/CDS	
SRA:SRR18779971.23685049.1	
Translation ORF/CDS	
SRA:SRR18779969.8691412.2	
Translation ORF/CDS	
SRA:SRR18779971.4811095.2	CTGGAAGGTTCCAAGGAGAAAAAACATCACCACCAAATAATAATTCAGTGAGA
Translation ORF/CDS	P G R F Q G E K T S P P N N N S V R
SRA:SRR18779971.11429211.2	CTGGAAGGTTCCAAGGAGAAAAAACATCACCACCAAATAATAATTCAGTGAGAGGTTATCAAGTA
Translation ORF/CDS	P G R F Q G E K T S P P N N N S V R G Y Q V
SRA:SRR18779971.22392603.1	CTGGAAGGTTCCAAGGAGAAAAAACATCACCACCAAATAATAATTCAGTGAGAGGTTATCAAGTAATTGA
Translation ORF/CDS	P G R F Q G E K T S P P N N N S V R G Y Q V I XX
SRA:SRR18779971.23396592.1	CTGGAAGGTTCCAAGGAGAAAAAACATCACCACCAAATAATAATTCAGTGAGAGGTTATCAAGTAATTGA
Translation ORF/CDS	P G R F Q G E K T S P P N N N S V R G Y Q V I D
SRA:SRR18779971.22392603.2	AGTAATTGA
Translation ORF/CDS	X V I D
SRA:SRR18779971.21820934.1	
Translation ORF/CDS	
SRA:SRR18779971.21621599.2	
Translation ORF/CDS	
SRA:SRR18779971.23641517.2	
Translation ORF/CDS	
SRA:SRR18779971.21621599.1	
Translation ORF/CDS	
SRA:SRR18779971.21820934.2	
Translation ORF/CDS	
SRA:SRR18779971.21291262.1	
Translation ORF/CDS	
SRA:SRR18779971.23641517.1	
Translation ORF/CDS	
SRA:SRR18779971.21291262.2	
Translation ORF/CDS	
SRA:SRR18779970.2541555.2	
Translation ORF/CDS	
SRA:SRR18779971.8085033.2	
Translation ORF/CDS	

LOC101265511

360 380 400 420

XM_004234090 SOLLC mRNA_LOC101265511		TCAAGCTAAACAAAGGATTAAAACTATGTGCCCTGGGGCTGCTGTTTCTTGCGCTGATATTCTTGCCCTT
Translation ORF/CDS		Q A K Q R I K T M C P G A A V S C A D I L A L
Consensus		TCAAGCTAAACAAAGGATTAAAACTATGTGCCCTGGGGCTGCTGTTTCTTGCGCTGATATTCTTGCCCTT
Translation ORF/CDS		Q A K Q R I K T M C P G A A V S C A D I L A L
SRA:SRR18779971.23685049.2		
Translation ORF/CDS		
SRA:SRR18779971.23685049.1		
Translation ORF/CDS		
SRA:SRR18779969.8691412.2		
Translation ORF/CDS		
SRA:SRR18779971.4811095.2		
Translation ORF/CDS		
SRA:SRR18779971.11429211.2		
Translation ORF/CDS		
SRA:SRR18779971.22392603.1		
Translation ORF/CDS		
SRA:SRR18779971.23396592.1		TCAAGCTAAACAAAGGATTAAAACTATGTGCCCTGGGGCTGCTGTTTCTTGCGCTGATATTCTTGCCCTT
Translation ORF/CDS		Q A K Q R I K T M C P G A A V S C A D I L A L
SRA:SRR18779971.22392603.2		TCAAGCTAAACAAAGGATTAAAACTATGTGCCCTGGGGCTGCTGTTTCTTGCGCTGATATTCTTGCCCTT
Translation ORF/CDS		Q A K Q R I K T M C P G A A V S C A D I L A L
SRA:SRR18779971.21820934.1		GTTTCTTGCGCTGATATTCTTGCCCTT
Translation ORF/CDS		V S C A D I L A L
SRA:SRR18779971.21621599.2		
Translation ORF/CDS		
SRA:SRR18779971.23641517.2		
Translation ORF/CDS		
SRA:SRR18779971.21621599.1		
Translation ORF/CDS		
SRA:SRR18779971.21820934.2		
Translation ORF/CDS		
SRA:SRR18779971.21291262.1		
Translation ORF/CDS		
SRA:SRR18779971.23641517.1		
Translation ORF/CDS		
SRA:SRR18779971.21291262.2		
Translation ORF/CDS		
SRA:SRR18779970.2541555.2		
Translation ORF/CDS		
SRA:SRR18779971.8085033.2		
Translation ORF/CDS		

LOC101265511

440 460 480

XM_004234090 SOLLC mRNA_LOC101265511	GCTGCTCGTGACTCTGTTGCTATGTTAGGGGGAATTCCATACCCTGTGAGTCTAGGCCGGAGAGATGCAA
Translation ORF/CDS	A A R D S V A M L G G I P Y P V S L G R R D A
Consensus	GCTGCTCGTGACTCTGTTGCTATGTTAGGGGGAATTCCATACCCTGTGAGTCTAGGCCGGAGAGATGCAA
Translation ORF/CDS	A A R D S V A M L G G I P Y P V S L G R R D A
SRA:SRR18779971.23685049.2	
Translation ORF/CDS	
SRA:SRR18779971.23685049.1	
Translation ORF/CDS	
SRA:SRR18779969.8691412.2	
Translation ORF/CDS	
SRA:SRR18779971.4811095.2	
Translation ORF/CDS	
SRA:SRR18779971.11429211.2	
Translation ORF/CDS	
SRA:SRR18779971.22392603.1	
Translation ORF/CDS	
SRA:SRR18779971.23396592.1	G
Translation ORF/CDS	X
SRA:SRR18779971.22392603.2	GCTGCTCGTGACTCTGTTGCTATGTTAGGGGGAATTCCATACCCTGTGAGTCTAGGCCGGAGAGATGCAA
Translation ORF/CDS	A A R D S V A M L G G I P Y P V S L G R R D A
SRA:SRR18779971.21820934.1	GCTGCTCGTGACTCTGTTGCTATGTTAGGGGGAATTCCATACCCTGTGAGTCTAGGCCGGAGAGATGCAA
Translation ORF/CDS	A A R D S V A M L G G I P Y P V S L G R R D A
SRA:SRR18779971.21621599.2	TCTGTTGCTATGTTAGGGGGAATTCCATACCCTGTGAGTCTAGGCCGGAGAGATGCAA
Translation ORF/CDS	S V A M L G G I P Y P V S L G R R D A
SRA:SRR18779971.23641517.2	
Translation ORF/CDS	
SRA:SRR18779971.21621599.1	
Translation ORF/CDS	
SRA:SRR18779971.21820934.2	
Translation ORF/CDS	
SRA:SRR18779971.21291262.1	
Translation ORF/CDS	
SRA:SRR18779971.23641517.1	
Translation ORF/CDS	
SRA:SRR18779971.21291262.2	
Translation ORF/CDS	
SRA:SRR18779970.2541555.2	
Translation ORF/CDS	
SRA:SRR18779971.8085033.2	
Translation ORF/CDS	

LOC101265511

500 520 540 560

Sequence	Alignment / Translation ORF/CDS
XM_004234090 SOLLC mRNA_LOC101265511	GGACCGCGAATTTCACCGGGGCGTTAACTCAACTTCCAGCCCCATTCGACGATCTCAACGTCCAATTAAA
Translation ORF/CDS	R T A N F T G A L T Q L P A P F D D L N V Q L K
Consensus	GGACCGCGAATTTCACCGGGGCGTTAACTCAACTTCCAGCCCCATTCGACGATCTCAACGTCCAATTAAA
Translation ORF/CDS	R T A N F T G A L T Q L P A P F D D L N V Q L K
SRA:SRR18779971.23685049.2	
Translation ORF/CDS	
SRA:SRR18779971.23685049.1	
Translation ORF/CDS	
SRA:SRR18779969.8691412.2	
Translation ORF/CDS	
SRA:SRR18779971.4811095.2	
Translation ORF/CDS	
SRA:SRR18779971.11429211.2	
Translation ORF/CDS	
SRA:SRR18779971.22392603.1	
Translation ORF/CDS	
SRA:SRR18779971.23396592.1	
Translation ORF/CDS	
SRA:SRR18779971.22392603.2	G
Translation ORF/CDS	X X
SRA:SRR18779971.21820934.1	GGACCGCGAATTTCACCGGGGCGTTAACTCAACTTCCAGCCCCATTCGACGAT
Translation ORF/CDS	R T A N F T G A L T Q L P A P F D D
SRA:SRR18779971.21621599.2	GGACCGCGAATTTCACCGGGGCGTTAACTCAACTTCCAGCCCCATTCGACGATCTCAACGTCCAATTAAA
Translation ORF/CDS	R T A N F T G A L T Q L P A P F D D L N V Q L K
SRA:SRR18779971.23641517.2	CGCGAATTTCACCGGGGCGTTAACTCAACTTCCAGCCCCATTCGACGATCTCAACGTCCAATTAAA
Translation ORF/CDS	X A N F T G A L T Q L P A P F D D L N V Q L K
SRA:SRR18779971.21621599.1	CTTCCAGCCCCATTCGACGATCTCAACGTCCAATTAAA
Translation ORF/CDS	L P A P F D D L N V Q L K
SRA:SRR18779971.21820934.2	GATCTCAACGTCCAATTAAA
Translation ORF/CDS	D L N V Q L K
SRA:SRR18779971.21291262.1	
Translation ORF/CDS	
SRA:SRR18779971.23641517.1	
Translation ORF/CDS	
SRA:SRR18779971.21291262.2	
Translation ORF/CDS	
SRA:SRR18779970.2541555.2	
Translation ORF/CDS	
SRA:SRR18779971.8085033.2	
Translation ORF/CDS	

LOC101265511

580 600 620

XM_004234090 SOLLC mRNA_LOC101265511	AAAATTCAGCAATAAGGGAATGTCTCCTCGGGAAATGGTTGCGTTAGTCGGGTCCCACACAGTAGGATTT
Translation ORF/CDS	K F S N K G M S P R E M V A L V G S H T V G F
Consensus	AAAATTCAGCAATAAGGGAATGTCTCCTCGGGAAATGGTTGCGTTAGTCGGGTCCCACACAGTAGGATTT
Translation ORF/CDS	K F S N K G M S P R E M V A L V G S H T V G F
SRA:SRR18779971.23685049.2	
Translation ORF/CDS	
SRA:SRR18779971.23685049.1	
Translation ORF/CDS	
SRA:SRR18779969.8691412.2	
Translation ORF/CDS	
SRA:SRR18779971.4811095.2	
Translation ORF/CDS	
SRA:SRR18779971.11429211.2	
Translation ORF/CDS	
SRA:SRR18779971.22392603.1	
Translation ORF/CDS	
SRA:SRR18779971.23396592.1	
Translation ORF/CDS	
SRA:SRR18779971.22392603.2	
Translation ORF/CDS	
SRA:SRR18779971.21820934.1	
Translation ORF/CDS	
SRA:SRR18779971.21621599.2	AAAATTCAGCAATAAGGGAATG
Translation ORF/CDS	K F S N K G M
SRA:SRR18779971.23641517.2	AAAATTCAGCAATAAGGGAATGTCTCCTCGGGAAATGGTTGCGTTAGTCGGGTCCCACACAGTAGGATTT
Translation ORF/CDS	K F S N K G M S P R E M V A L V G S H T V G F
SRA:SRR18779971.21621599.1	AAAATTCAGCAATAAGGGAATGTCTCCTCGGGAAATGGTTGCGTTAGTCGGGTCCCACACAGTAGGATTT
Translation ORF/CDS	K F S N K G M S P R E M V A L V G S H T V G F
SRA:SRR18779971.21820934.2	AAAATTCAGCAATAAGGGAATGTCTCCTCGGGAAATGGTTGCGTTAGTCGGGTCCCACACAGTAGGATTT
Translation ORF/CDS	K F S N K G M S P R E M V A L V G S H T V G F
SRA:SRR18779971.21291262.1	TAGTCGGGTCCCACACAGTAGGATTT
Translation ORF/CDS	XX V G S H T V G F
SRA:SRR18779971.23641517.1	ACAGTAGGATTT
Translation ORF/CDS	T V G F
SRA:SRR18779971.21291262.2	
Translation ORF/CDS	
SRA:SRR18779970.2541555.2	
Translation ORF/CDS	
SRA:SRR18779971.8085033.2	
Translation ORF/CDS	

LOC101265511

640 660 680 700

XM_004234090 SOLLC mRNA_LOC101265511	GCACGATGTGTGACATTATGTGACGACAGAAACATCAACCCGGCCATGAAATCAACACTAAAATGCGGTT
Translation ORF/CDS	A R C V T L C D D R N I N P A M K S T L K C G
Consensus	GCACGATGTGTGACATTATGTGACGACAGAAACATCAACCCGGCCATGAAATCAACACTAAAATGCGGTT
Translation ORF/CDS	A R C V T L C D D R N I N P A M K S T L K C G
SRA:SRR18779971.23685049.2	
Translation ORF/CDS	
SRA:SRR18779971.23685049.1	
Translation ORF/CDS	
SRA:SRR18779969.8691412.2	
Translation ORF/CDS	
SRA:SRR18779971.4811095.2	
Translation ORF/CDS	
SRA:SRR18779971.11429211.2	
Translation ORF/CDS	
SRA:SRR18779971.22392603.1	
Translation ORF/CDS	
SRA:SRR18779971.23396592.1	
Translation ORF/CDS	
SRA:SRR18779971.22392603.2	
Translation ORF/CDS	
SRA:SRR18779971.21820934.1	
Translation ORF/CDS	
SRA:SRR18779971.21621599.2	
Translation ORF/CDS	
SRA:SRR18779971.23641517.2	GCACGATGTGTGAC
Translation ORF/CDS	A R C V XX
SRA:SRR18779971.21621599.1	GCACGATGTGTGACATTATGTGACGACAGAAACATCAACCCG
Translation ORF/CDS	A R C V T L C D D R N I N P
SRA:SRR18779971.21820934.2	GCACGATGTGTGACATTATGTGACGACAGAAACATCAACCCGGCCATGAAATCAACACTA
Translation ORF/CDS	A R C V T L C D D R N I N P A M K S T L
SRA:SRR18779971.21291262.1	GCACGATGTGTGACATTATGTGACGACAGAAACATCAACCCGGCCATGAAATCAACACTAAAATGCGGTT
Translation ORF/CDS	A R C V T L C D D R N I N P A M K S T L K C G
SRA:SRR18779971.23641517.1	GCACGATGTGTGACATTATGTGACGACAGAAACATCAACCCGGCCATGAAATCAACACTAAAATGCGGTT
Translation ORF/CDS	A R C V T L C D D R N I N P A M K S T L K C G
SRA:SRR18779971.21291262.2	
Translation ORF/CDS	
SRA:SRR18779970.2541555.2	
Translation ORF/CDS	
SRA:SRR18779971.8085033.2	
Translation ORF/CDS	

LOC101265511

720 740 760

XM_004234090 SOLLC mRNA_LOC101265511 GCCCTGTTAGTAACAATAACACAAACTTAGTACCATTGGATTTAATGACTCCTGATTTTTTCGATAAGTT
Translation ORF/CDS C P V S N N N T N L V P L D L M T P D F F D K F

Consensus GCCCTGTTAGTAACAATAACACAAACTTAGTACCATTGGATTTAATGACTCCTGATTTTTTCGATAAGTT
Translation ORF/CDS C P V S N N N T N L V P L D L M T P D F F D K F

SRA:SRR18779971.23685049.2
Translation ORF/CDS

SRA:SRR18779971.23685049.1
Translation ORF/CDS

SRA:SRR18779969.8691412.2
Translation ORF/CDS

SRA:SRR18779971.4811095.2
Translation ORF/CDS

SRA:SRR18779971.11429211.2
Translation ORF/CDS

SRA:SRR18779971.22392603.1
Translation ORF/CDS

SRA:SRR18779971.23396592.1
Translation ORF/CDS

SRA:SRR18779971.22392603.2
Translation ORF/CDS

SRA:SRR18779971.21820934.1
Translation ORF/CDS

SRA:SRR18779971.21621599.2
Translation ORF/CDS

SRA:SRR18779971.23641517.2
Translation ORF/CDS

SRA:SRR18779971.21621599.1
Translation ORF/CDS

SRA:SRR18779971.21820934.2
Translation ORF/CDS

SRA:SRR18779971.21291262.1 GCCCTGTTAGTAACAATAACACAAACTTAGTACCATTGGATTTAATGACTCCTG
Translation ORF/CDS C P V S N N N T N L V P L D L M T P X

SRA:SRR18779971.23641517.1 GCCCTGTTAGTAACAATAACACAAACTTAGTACCATTGGATTTAATGACTCCTGATTTTTTCGATAAG
Translation ORF/CDS C P V S N N N T N L V P L D L M T P D F F D K

SRA:SRR18779971.21291262.2 ATTTAATGACTCCTGATTTTTTCGATAAGTT
Translation ORF/CDS XX L M T P D F F D K F

SRA:SRR18779970.2541555.2
Translation ORF/CDS

SRA:SRR18779971.8085033.2
Translation ORF/CDS

LOC101265511

780 800 820 840

XM_004234090 SOLLC mRNA_LOC101265511	TTATTACGAGGATTTAATTCGAAATCAAGGACTTCTTTTCTCAGACCAAGTATTGATGGGATCTACCGCG
Translation ORF/CDS	Y Y E D L I R N Q G L L F S D Q V L M G S T A
Consensus	TTATTACGAGGATTTAATTCGAAATCAAGGACTTCTTTTCTCAGACCAAGTATTGATGGGATCTACCGCG
Translation ORF/CDS	Y Y E D L I R N Q G L L F S D Q V L M G S T A
SRA:SRR18779971.23685049.2	
Translation ORF/CDS	
SRA:SRR18779971.23685049.1	
Translation ORF/CDS	
SRA:SRR18779969.8691412.2	
Translation ORF/CDS	
SRA:SRR18779971.4811095.2	
Translation ORF/CDS	
SRA:SRR18779971.11429211.2	
Translation ORF/CDS	
SRA:SRR18779971.22392603.1	
Translation ORF/CDS	
SRA:SRR18779971.23396592.1	
Translation ORF/CDS	
SRA:SRR18779971.22392603.2	
Translation ORF/CDS	
SRA:SRR18779971.21820934.1	
Translation ORF/CDS	
SRA:SRR18779971.21621599.2	
Translation ORF/CDS	
SRA:SRR18779971.23641517.2	
Translation ORF/CDS	
SRA:SRR18779971.21621599.1	
Translation ORF/CDS	
SRA:SRR18779971.21820934.2	
Translation ORF/CDS	
SRA:SRR18779971.21291262.1	
Translation ORF/CDS	
SRA:SRR18779971.23641517.1	
Translation ORF/CDS	
SRA:SRR18779971.21291262.2	TTATTACGAGGATTTAATTCGAAATCAAGGACTTCTTTTCTCAGACCAAGTATTGATGGGATCTACCGCG
Translation ORF/CDS	Y Y E D L I R N Q G L L F S D Q V L M G S T A
SRA:SRR18779970.2541555.2	
Translation ORF/CDS	
SRA:SRR18779971.8085033.2	
Translation ORF/CDS	

LOC101265511

860 880 900

XM_004234090 SOLLC mRNA_LOC101265511 ACTAGTGATGTTGTTCGGACCTATAATAGTAACCCTACTCTATTTTTACGCGAATTCAACGATGCCATGA
Translation ORF/CDS T S D V V R T Y N S N P T L F L R E F N D A M

Consensus ACTAGTGATGTTGTTCGGACCTATAATAGTAACCCTACTCTATTTTTACGCGAATTCAACGATGCCATGA
Translation ORF/CDS T S D V V R T Y N S N P T L F L R E F N D A M

SRA:SRR18779971.23685049.2
Translation ORF/CDS

SRA:SRR18779971.23685049.1
Translation ORF/CDS

SRA:SRR18779969.8691412.2
Translation ORF/CDS

SRA:SRR18779971.4811095.2
Translation ORF/CDS

SRA:SRR18779971.11429211.2
Translation ORF/CDS

SRA:SRR18779971.22392603.1
Translation ORF/CDS

SRA:SRR18779971.23396592.1
Translation ORF/CDS

SRA:SRR18779971.22392603.2
Translation ORF/CDS

SRA:SRR18779971.21820934.1
Translation ORF/CDS

SRA:SRR18779971.21621599.2
Translation ORF/CDS

SRA:SRR18779971.23641517.2
Translation ORF/CDS

SRA:SRR18779971.21621599.1
Translation ORF/CDS

SRA:SRR18779971.21820934.2
Translation ORF/CDS

SRA:SRR18779971.21291262.1
Translation ORF/CDS

SRA:SRR18779971.23641517.1
Translation ORF/CDS

SRA:SRR18779971.21291262.2 ACTAGTGATGTTGTTCGGACCTATAATAGTAACCCTACTCTATTTTTAC
Translation ORF/CDS T S D V V R T Y N S N P T L F L X

SRA:SRR18779970.2541555.2 GTTGTTCGGACCTATAATAGTAACCCTACTCTATTTTTACGCGAATTCAACGATGCCATGA
Translation ORF/CDS V V R T Y N S N P T L F L R E F N D A M

SRA:SRR18779971.8085033.2 TGA
Translation ORF/CDS XX

LOC101265511

920 940 960 980

XM_004234090 SOLLC mRNA_LOC101265511	TAAAGATGGGGAACTTGCCCCCATCTCGTGGCGTTCAATTGGAAATTCGCGATGTTTGTAGCAAGGTGAA
Translation ORF/CDS	I K M G N L P P S R G V Q L E I R D V C S K V N
Consensus	TAAAGATGGGGAACTTGCCCCCATCTCGTGGCGTTCAATTGGAAATTCGCGATGTTTGTAGCAAGGTGAA
Translation ORF/CDS	I K M G N L P P S R G V Q L E I R D V C S K V N
SRA:SRR18779971.23685049.2	
Translation ORF/CDS	
SRA:SRR18779971.23685049.1	
Translation ORF/CDS	
SRA:SRR18779969.8691412.2	
Translation ORF/CDS	
SRA:SRR18779971.4811095.2	
Translation ORF/CDS	
SRA:SRR18779971.11429211.2	
Translation ORF/CDS	
SRA:SRR18779971.22392603.1	
Translation ORF/CDS	
SRA:SRR18779971.23396592.1	
Translation ORF/CDS	
SRA:SRR18779971.22392603.2	
Translation ORF/CDS	
SRA:SRR18779971.21820934.1	
Translation ORF/CDS	
SRA:SRR18779971.21621599.2	
Translation ORF/CDS	
SRA:SRR18779971.23641517.2	
Translation ORF/CDS	
SRA:SRR18779971.21621599.1	
Translation ORF/CDS	
SRA:SRR18779971.21820934.2	
Translation ORF/CDS	
SRA:SRR18779971.21291262.1	
Translation ORF/CDS	
SRA:SRR18779971.23641517.1	
Translation ORF/CDS	
SRA:SRR18779971.21291262.2	
Translation ORF/CDS	
SRA:SRR18779970.2541555.2	TAAAGATGGGGAACTTGCCCCCATCTCGTGGCGTTCAATTGGAAATTCGCGATGTTTGTAGCAAGGTGAA
Translation ORF/CDS	I K M G N L P P S R G V Q L E I R D V C S K V N
SRA:SRR18779971.8085033.2	TAAAGATGGGGAACTTGCCCCCATCTCGTGGCGTTCAATTGGAAATTCGCGATGTTTGTAGCAAGGTGAA
Translation ORF/CDS	I K M G N L P P S R G V Q L E I R D V C S K V N

LOC101265511

1,000

XM_004234090 SOLLC mRNA_LOC101265511	TAGTAATTCTATCGCGGACATGTAA
Translation ORF/CDS	S N S I A D M *
Consensus	TAGTAATTCTATCGCGGACATGTAA
Translation ORF/CDS	S N S I A D M *
SRA:SRR18779971.23685049.2	
Translation ORF/CDS	
SRA:SRR18779971.23685049.1	
Translation ORF/CDS	
SRA:SRR18779969.8691412.2	
Translation ORF/CDS	
SRA:SRR18779971.4811095.2	
Translation ORF/CDS	
SRA:SRR18779971.11429211.2	
Translation ORF/CDS	
SRA:SRR18779971.22392603.1	
Translation ORF/CDS	
SRA:SRR18779971.23396592.1	
Translation ORF/CDS	
SRA:SRR18779971.22392603.2	
Translation ORF/CDS	
SRA:SRR18779971.21820934.1	
Translation ORF/CDS	
SRA:SRR18779971.21621599.2	
Translation ORF/CDS	
SRA:SRR18779971.23641517.2	
Translation ORF/CDS	
SRA:SRR18779971.21621599.1	
Translation ORF/CDS	
SRA:SRR18779971.21820934.2	
Translation ORF/CDS	
SRA:SRR18779971.21291262.1	
Translation ORF/CDS	
SRA:SRR18779971.23641517.1	
Translation ORF/CDS	
SRA:SRR18779971.21291262.2	
Translation ORF/CDS	
SRA:SRR18779970.2541555.2	TAGTAATTCTATCGCGGAC
Translation ORF/CDS	S N S I A D
SRA:SRR18779971.8085033.2	TAGTAATTCTATCGCGGACATGTAA
Translation ORF/CDS	S N S I A D M *
