# Supplementary material for: A new versatile peroxidase with extremophilic traits over-produced in MicroTom cell cultures
Source: Sci Rep. 2023 Sep 15;13:15338. doi: 10.1038/s41598-023-42597-x (PMC10504257; doi:10.1038/s41598-023-42597-x)
Supplement: Supplementary file 2 — Supplementary Figure 2. [file 41598_2023_42597_MOESM2_ESM.pdf]

Suppl. Fig. B. Blastn analysis of whole genome shotgun sequences of cultivar Micro-Tom chromosome 3, using the cDNA of SAAP2 reference gene as a query. Differences in sequences (conflicts) are indicated for both nucleotides and translations.

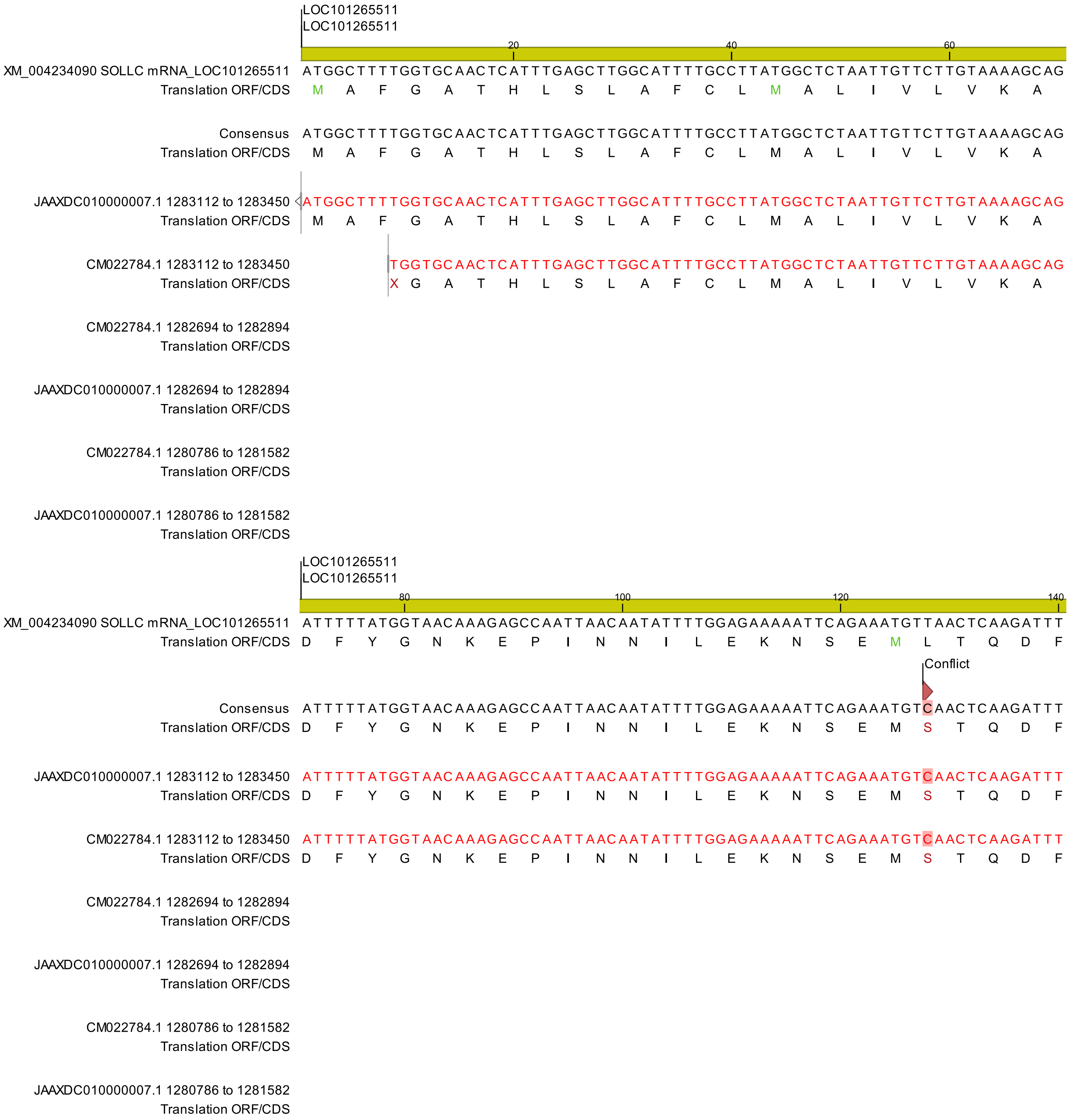

```
                                      LOC101265511
                                      LOC101265511
                                                          160                    180                    200
XM_004234090 SOLLC mRNA_LOC101265511  TTGTATTTTCGCGGCGGTTGGATCTGTTGTGACGGAAGCTATTGCAAGGGAGAGGCGAATGGGAGCGTCT
                   Translation ORF/CDS    C  I  F  A  A  V  G  S  V  V  T  E  A  I  A  R  E  R  R  M  G  A  S

                            Consensus  TTGTATTTTCGCGGCGGTTGGATCTGTTGTGACGGAAGCTATTGCAAGGGAGAGGCGAATGGGAGCGTCT
                   Translation ORF/CDS    C  I  F  A  A  V  G  S  V  V  T  E  A  I  A  R  E  R  R  M  G  A  S

   JAAXDC010000007.1 1283112 to 1283450  TTGTATTTTCGCGGCGGTTGGATCTGTTGTGACGGAAGCTATTGCAAGGGAGAGGCGAATGGGAGCGTCT
                   Translation ORF/CDS    C  I  F  A  A  V  G  S  V  V  T  E  A  I  A  R  E  R  R  M  G  A  S

          CM022784.1 1283112 to 1283450  TTGTATTTTCGCGGCGGTTGGATCTGTTGTGACGGAAGCTATTGCAAGGGAGAGGCGAATGGGAGCGTCT
                   Translation ORF/CDS    C  I  F  A  A  V  G  S  V  V  T  E  A  I  A  R  E  R  R  M  G  A  S

          CM022784.1 1282694 to 1282894
                   Translation ORF/CDS

   JAAXDC010000007.1 1282694 to 1282894
                   Translation ORF/CDS

          CM022784.1 1280786 to 1281582
                   Translation ORF/CDS

   JAAXDC010000007.1 1280786 to 1281582
                   Translation ORF/CDS

                                      LOC101265511
                                      LOC101265511
                                                 220                    240                    260                  280
XM_004234090 SOLLC mRNA_LOC101265511  CTCATTCGCCTCTTCTTCCACGACTGCTTTGTCGATGGATGTGATGCTGGAATTCTTCTAGATGATATTC
                   Translation ORF/CDS   L  I  R  L  F  F  H  D  C  F  V  D  G  C  D  A  G  I  L  L  D  D  I

                            Consensus  CTCATTCGCCTCTTCTTCCACGACTGCTTTGTCGATGGATGTGATGCTGGAATTCTTCTAGATGATATTC
                   Translation ORF/CDS   L  I  R  L  F  F  H  D  C  F  V  D  G  C  D  A  G  I  L  L  D  D  I

   JAAXDC010000007.1 1283112 to 1283450  CTCATTCGCCTCTTCTTCCACGACTGCTTTGTCGATG
                   Translation ORF/CDS   L  I  R  L  F  F  H  D  C  F  V  D X

          CM022784.1 1283112 to 1283450  CTCATTCGCCTCTTCTTCCACGACTGCTTTGTCGATG
                   Translation ORF/CDS   L  I  R  L  F  F  H  D  C  F  V  D X

          CM022784.1 1282694 to 1282894                                      GGATGTGATGCTGGAATTCTTCTAGATGATATTC
                   Translation ORF/CDS                                        G  C  D  A  G  I  L  L  D  D  I

   JAAXDC010000007.1 1282694 to 1282894                                      GGATGTGATGCTGGAATTCTTCTAGATGATATTC
                   Translation ORF/CDS                                        G  C  D  A  G  I  L  L  D  D  I

          CM022784.1 1280786 to 1281582
                   Translation ORF/CDS

   JAAXDC010000007.1 1280786 to 1281582
                   Translation ORF/CDS
```

LOC101265511
LOC101265511
300 320 340

XM_004234090 SOLLC mRNA_LOC101265511 CTGGAAGGTTCCAAGGAGAAAAAACATCACCACCAAATAATAATTCAGTGAGAGGTTATCAAGTAATTGA
Translation ORF/CDS P G R F Q G E K T S P P N N N S V R G Y Q V I D

Consensus CTGGAAGGTTCCAAGGAGAAAAAACATCACCACCAAATAATAATTCAGTGAGAGGTTATCAAGTAATTGA
Translation ORF/CDS P G R F Q G E K T S P P N N N S V R G Y Q V I D

JAAXDC010000007.1 1283112 to 1283450
Translation ORF/CDS

CM022784.1 1283112 to 1283450
Translation ORF/CDS

CM022784.1 1282694 to 1282894 CTGGAAGGTTCCAAGGAGAAAAAACATCACCACCAAATAATAATTCAGTGAGAGGTTATCAAGTAATTGA
Translation ORF/CDS P G R F Q G E K T S P P N N N S V R G Y Q V I D

JAAXDC010000007.1 1282694 to 1282894 CTGGAAGGTTCCAAGGAGAAAAAACATCACCACCAAATAATAATTCAGTGAGAGGTTATCAAGTAATTGA
Translation ORF/CDS P G R F Q G E K T S P P N N N S V R G Y Q V I D

CM022784.1 1280786 to 1281582
Translation ORF/CDS

JAAXDC010000007.1 1280786 to 1281582
Translation ORF/CDS

LOC101265511
LOC101265511
360 380 400 420

XM_004234090 SOLLC mRNA_LOC101265511 TCAAGCTAAACAAAGGATTAAAACTATGTGCCCTGGGGCTGCTGTTTCTTGCGCTGATATTCTTGCCCTT
Translation ORF/CDS Q A K Q R I K T M C P G A A V S C A D I L A L

Consensus TCAAGCTAAACAAAGGATTAAAACTATGTGCCCTGGGGCTGCTGTTTCTTGCGCTGATATTCTTGCCCTT
Translation ORF/CDS Q A K Q R I K T M C P G A A V S C A D I L A L

JAAXDC010000007.1 1283112 to 1283450
Translation ORF/CDS

CM022784.1 1283112 to 1283450
Translation ORF/CDS

CM022784.1 1282694 to 1282894 TCAAGCTAAACAAAGGATTAAAACTATGTGCCCTGGGGCTGCTGTTTCTTGCGCTGATATTCTTGCCCTT
Translation ORF/CDS Q A K Q R I K T M C P G A A V S C A D I L A L

JAAXDC010000007.1 1282694 to 1282894 TCAAGCTAAACAAAGGATTAAAACTATGTGCCCTGGGGCTGCTGTTTCTTGCGCTGATATTCTTGCCCTT
Translation ORF/CDS Q A K Q R I K T M C P G A A V S C A D I L A L

CM022784.1 1280786 to 1281582
Translation ORF/CDS

JAAXDC010000007.1 1280786 to 1281582
Translation ORF/CDS

```
                                        LOC101265511
                                        LOC101265511
                                                      440                        460                        480
XM_004234090 SOLLC mRNA_LOC101265511   GCTGCTCGTGACTCTGTTGCTATGTTAGGGGGAATTCCATACCCTGTGAGTCTAGGCCGGAGAGATGCAA
                Translation ORF/CDS      A  A  R  D  S  V  A  M  L  G  G  I  P  Y  P  V  S  L  G  R  R  D  A
                                                                                                   Conflict
                          Consensus    GCTGCTCGTGACTCTGTTGCTATGTTAGGGGGAATTCCATACCCTGTGAGTCTAGGCCGGAGAGACGCAA
                Translation ORF/CDS      A  A  R  D  S  V  A  M  L  G  G  I  P  Y  P  V  S  L  G  R  R  D  A

JAAXDC010000007.1 1283112 to 1283450
                Translation ORF/CDS

     CM022784.1 1283112 to 1283450
                Translation ORF/CDS

     CM022784.1 1282694 to 1282894     GCTGCTCGTGACTCTGTTGCTATGGTA
                Translation ORF/CDS      A  A  R  D  S  V  A  M  V

JAAXDC010000007.1 1282694 to 1282894   GCTGCTCGTGACTCTGTTGCTATGGTA
                Translation ORF/CDS      A  A  R  D  S  V  A  M  V

     CM022784.1 1280786 to 1281582                             GTTAGGGGGAATTCCATACCCTGTGAGTCTAGGCCGGAGAGACGCAA
                Translation ORF/CDS                            X L  G  G  I  P  Y  P  V  S  L  G  R  R  D  A

JAAXDC010000007.1 1280786 to 1281582                           GTTAGGGGGAATTCCATACCCTGTGAGTCTAGGCCGGAGAGACGCAA
                Translation ORF/CDS                            X L  G  G  I  P  Y  P  V  S  L  G  R  R  D  A

                                        LOC101265511
                                        LOC101265511
                                                 500                        520                        540                 560
XM_004234090 SOLLC mRNA_LOC101265511   GGACCGCGAATTTCACCGGGGCGTTAACTCAACTTCCAGCCCCATTCGACGATCTCAACGTCCAATTAAA
                Translation ORF/CDS    R  T  A  N  F  T  G  A  L  T  Q  L  P  A  P  F  D  D  L  N  V  Q  L  K
                                               Conflict
                          Consensus    GGACCGCAAATTTCACCGGGGCGTTAACTCAACTTCCAGCCCCATTCGACGATCTCAACGTCCAATTAAA
                Translation ORF/CDS    R  T  A  N  F  T  G  A  L  T  Q  L  P  A  P  F  D  D  L  N  V  Q  L  K

JAAXDC010000007.1 1283112 to 1283450
                Translation ORF/CDS

     CM022784.1 1283112 to 1283450
                Translation ORF/CDS

     CM022784.1 1282694 to 1282894
                Translation ORF/CDS

JAAXDC010000007.1 1282694 to 1282894
                Translation ORF/CDS

     CM022784.1 1280786 to 1281582     GGACCGCAAATTTCACCGGGGCGTTAACTCAACTTCCAGCCCCATTCGACGATCTCAACGTCCAATTAAA
                Translation ORF/CDS    R  T  A  N  F  T  G  A  L  T  Q  L  P  A  P  F  D  D  L  N  V  Q  L  K

JAAXDC010000007.1 1280786 to 1281582   GGACCGCAAATTTCACCGGGGCGTTAACTCAACTTCCAGCCCCATTCGACGATCTCAACGTCCAATTAAA
                Translation ORF/CDS    R  T  A  N  F  T  G  A  L  T  Q  L  P  A  P  F  D  D  L  N  V  Q  L  K
```

LOC101265511
LOC101265511

580 600 620

XM_004234090 SOLLC mRNA_LOC101265511 AAAATTCAGCAATAAGGGAATGTCTCCTCGGGAAATGGTTGCGTTAGTCGGGTCCCACACAGTAGGATTT
Translation ORF/CDS K F S N K G M S P R E M V A L V G S H T V G F

Conflict

Consensus AAAATTCAGCGATAAGGGAATGTCTCCTCGGGAAATGGTTGCGTTAGTCGGGTCCCACACAGTAGGATTT
Translation ORF/CDS K F S D K G M S P R E M V A L V G S H T V G F

JAAXDC010000007.1 1283112 to 1283450
Translation ORF/CDS

CM022784.1 1283112 to 1283450
Translation ORF/CDS

CM022784.1 1282694 to 1282894
Translation ORF/CDS

JAAXDC010000007.1 1282694 to 1282894
Translation ORF/CDS

CM022784.1 1280786 to 1281582 AAAATTCAGCGATAAGGGAATGTCTCCTCGGGAAATGGTTGCGTTAGTCGGGTCCCACACAGTAGGATTT
Translation ORF/CDS K F S D K G M S P R E M V A L V G S H T V G F

JAAXDC010000007.1 1280786 to 1281582 AAAATTCAGCGATAAGGGAATGTCTCCTCGGGAAATGGTTGCGTTAGTCGGGTCCCACACAGTAGGATTT
Translation ORF/CDS K F S D K G M S P R E M V A L V G S H T V G F

LOC101265511
LOC101265511

640 660 680 700

XM_004234090 SOLLC mRNA_LOC101265511 GCACGATGTGTGACATTATGTGACGACAGAAACATCAACCCGGCCATGAAATCAACACTAAAATGCGGTT
Translation ORF/CDS A R C V T L C D D R N I N P A M K S T L K C G

Consensus GCACGATGTGTGACATTATGTGACGACAGAAACATCAACCCGGCCATGAAATCAACACTAAAATGCGGTT
Translation ORF/CDS A R C V T L C D D R N I N P A M K S T L K C G

JAAXDC010000007.1 1283112 to 1283450
Translation ORF/CDS

CM022784.1 1283112 to 1283450
Translation ORF/CDS

CM022784.1 1282694 to 1282894
Translation ORF/CDS

JAAXDC010000007.1 1282694 to 1282894
Translation ORF/CDS

CM022784.1 1280786 to 1281582 GCACGATGTGTGACATTATGTGACGACAGAAACATCAACCCGGCCATGAAATCAACACTAAAATGCGGTT
Translation ORF/CDS A R C V T L C D D R N I N P A M K S T L K C G

JAAXDC010000007.1 1280786 to 1281582 GCACGATGTGTGACATTATGTGACGACAGAAACATCAACCCGGCCATGAAATCAACACTAAAATGCGGTT
Translation ORF/CDS A R C V T L C D D R N I N P A M K S T L K C G

```
                                          LOC101265511
                                          LOC101265511
                                                                     720                      740                      760
XM_004234090 SOLLC mRNA_LOC101265511  GCCCTGTTAGTAACAATAACACAAACTTAGTACCATTGGATTTAATGACTCCTGATTTTTTCGATAAGTT
                 Translation ORF/CDS  C  P  V  S  N  N  N  T  N  L  V  P  L  D  L  M  T  P  D  F  F  D  K  F

                           Consensus  GCCCTGTTAGTAACAATAACACAAACTTAGTACCATTGGATTTAATGACTCCTGATTTTTTCGATAAGTT
                 Translation ORF/CDS  C  P  V  S  N  N  N  T  N  L  V  P  L  D  L  M  T  P  D  F  F  D  K  F

JAAXDC010000007.1 1283112 to 1283450
                 Translation ORF/CDS

      CM022784.1 1283112 to 1283450
                 Translation ORF/CDS

      CM022784.1 1282694 to 1282894
                 Translation ORF/CDS

JAAXDC010000007.1 1282694 to 1282894
                 Translation ORF/CDS

      CM022784.1 1280786 to 1281582  GCCCTGTTAGTAACAATAACACAAACTTAGTACCATTGGATTTAATGACTCCTGATTTTTTCGATAAGTT
                 Translation ORF/CDS  C  P  V  S  N  N  N  T  N  L  V  P  L  D  L  M  T  P  D  F  F  D  K  F

JAAXDC010000007.1 1280786 to 1281582  GCCCTGTTAGTAACAATAACACAAACTTAGTACCATTGGATTTAATGACTCCTGATTTTTTCGATAAGTT
                 Translation ORF/CDS  C  P  V  S  N  N  N  T  N  L  V  P  L  D  L  M  T  P  D  F  F  D  K  F
```

```
                                          LOC101265511
                                          LOC101265511
                                                       780                      800                      820                840
XM_004234090 SOLLC mRNA_LOC101265511  TTATTACGAGGATTTAATTCGAAATCAAGGACTTCTTTTCTCAGACCAAGTATTGATGGGATCTACCGCG
                 Translation ORF/CDS    Y  Y  E  D  L  I  R  N  Q  G  L  L  F  S  D  Q  V  L  M  G  S  T  A

                           Consensus  TTATTACGAGGATTTAATTCGAAATCAAGGACTTCTTTTCTCAGACCAAGTATTGATGGGATCTACCGCG
                 Translation ORF/CDS    Y  Y  E  D  L  I  R  N  Q  G  L  L  F  S  D  Q  V  L  M  G  S  T  A

JAAXDC010000007.1 1283112 to 1283450
                 Translation ORF/CDS

      CM022784.1 1283112 to 1283450
                 Translation ORF/CDS

      CM022784.1 1282694 to 1282894
                 Translation ORF/CDS

JAAXDC010000007.1 1282694 to 1282894
                 Translation ORF/CDS

      CM022784.1 1280786 to 1281582  TTATTACGAGGATTTAATTCGAAATCAAGGACTTCTTTTCTCAGACCAAGTATTGATGGGATCTACCGCG
                 Translation ORF/CDS    Y  Y  E  D  L  I  R  N  Q  G  L  L  F  S  D  Q  V  L  M  G  S  T  A

JAAXDC010000007.1 1280786 to 1281582  TTATTACGAGGATTTAATTCGAAATCAAGGACTTCTTTTCTCAGACCAAGTATTGATGGGATCTACCGCG
                 Translation ORF/CDS    Y  Y  E  D  L  I  R  N  Q  G  L  L  F  S  D  Q  V  L  M  G  S  T  A
```

LOC101265511
LOC101265511

860 880 900

XM_004234090 SOLLC mRNA_LOC101265511 ACTAGTGATGTTGTTCGGACCTATAATAGTAACCCTACTCTATTTTTACGCGAATTCAACGATGCCATGA
Translation ORF/CDS T S D V V R T Y N S N P T L F L R E F N D A M

Consensus ACTAGTGATGTTGTTCGGACCTATAATAGTAACCCTACTCTATTTTTACGCGAATTCAACGATGCCATGA
Translation ORF/CDS T S D V V R T Y N S N P T L F L R E F N D A M

JAAXDC010000007.1 1283112 to 1283450
Translation ORF/CDS

CM022784.1 1283112 to 1283450
Translation ORF/CDS

CM022784.1 1282694 to 1282894
Translation ORF/CDS

JAAXDC010000007.1 1282694 to 1282894
Translation ORF/CDS

CM022784.1 1280786 to 1281582 ACTAGTGATGTTGTTCGGACCTATAATAGTAACCCTACTCTATTTTTACGCGAATTCAACGATGCCATGA
Translation ORF/CDS T S D V V R T Y N S N P T L F L R E F N D A M

JAAXDC010000007.1 1280786 to 1281582 ACTAGTGATGTTGTTCGGACCTATAATAGTAACCCTACTCTATTTTTACGCGAATTCAACGATGCCATGA
Translation ORF/CDS T S D V V R T Y N S N P T L F L R E F N D A M

LOC101265511
LOC101265511

920 940 960 980

XM_004234090 SOLLC mRNA_LOC101265511 TAAAGATGGGGAACTTGCCCCCATCTCGTGGCGTTCAATTGGAAATTCGCGATGTTTGTAGCAAGGTGAA
Translation ORF/CDS I K M G N L P P S R G V Q L E I R D V C S K V N

Consensus TAAAGATGGGGAACTTGCCCCCATCTCGTGGCGTTCAATTGGAAATTCGCGATGTTTGTAGCAAGGTGAA
Translation ORF/CDS I K M G N L P P S R G V Q L E I R D V C S K V N

JAAXDC010000007.1 1283112 to 1283450
Translation ORF/CDS

CM022784.1 1283112 to 1283450
Translation ORF/CDS

CM022784.1 1282694 to 1282894
Translation ORF/CDS

JAAXDC010000007.1 1282694 to 1282894
Translation ORF/CDS

CM022784.1 1280786 to 1281582 TAAAGATGGGGAACTTGCCCCCATCTCGTGGCGTTCAATTGGAAATTCGCGATGTTTGTAGCAAGGTGAA
Translation ORF/CDS I K M G N L P P S R G V Q L E I R D V C S K V N

JAAXDC010000007.1 1280786 to 1281582 TAAAGATGGGGAACTTGCCCCCATCTCGTGGCGTTCAATTGGAAATTCGCGATGTTTGTAGCAAGGTGAA
Translation ORF/CDS I K M G N L P P S R G V Q L E I R D V C S K V N

LOC101265511
LOC101265511
1,000

XM_004234090 SOLLC mRNA_LOC101265511 TAGTAATTCTATCGCGGACATGTAA
Translation ORF/CDS S N S I A D M *

Consensus TAGTAATTCTATCGCGGACATGTAA
Translation ORF/CDS S N S I A D M *

JAAXDC010000007.1 1283112 to 1283450
Translation ORF/CDS

CM022784.1 1283112 to 1283450
Translation ORF/CDS

CM022784.1 1282694 to 1282894
Translation ORF/CDS

JAAXDC010000007.1 1282694 to 1282894
Translation ORF/CDS

CM022784.1 1280786 to 1281582 TAGTAATTCTATCGCGGACATGTAA
Translation ORF/CDS S N S I A D M *

JAAXDC010000007.1 1280786 to 1281582 TAGTAATTCTATCGCGGACATGTAA
Translation ORF/CDS S N S I A D M *
