# Supplementary material for: A new versatile peroxidase with extremophilic traits over-produced in MicroTom cell cultures
Source: Sci Rep. 2023 Sep 15;13:15338. doi: 10.1038/s41598-023-42597-x (PMC10504257; doi:10.1038/s41598-023-42597-x)
Supplement: Supplementary file 3 — Supplementary Figure 3. [file 41598_2023_42597_MOESM3_ESM.pdf]

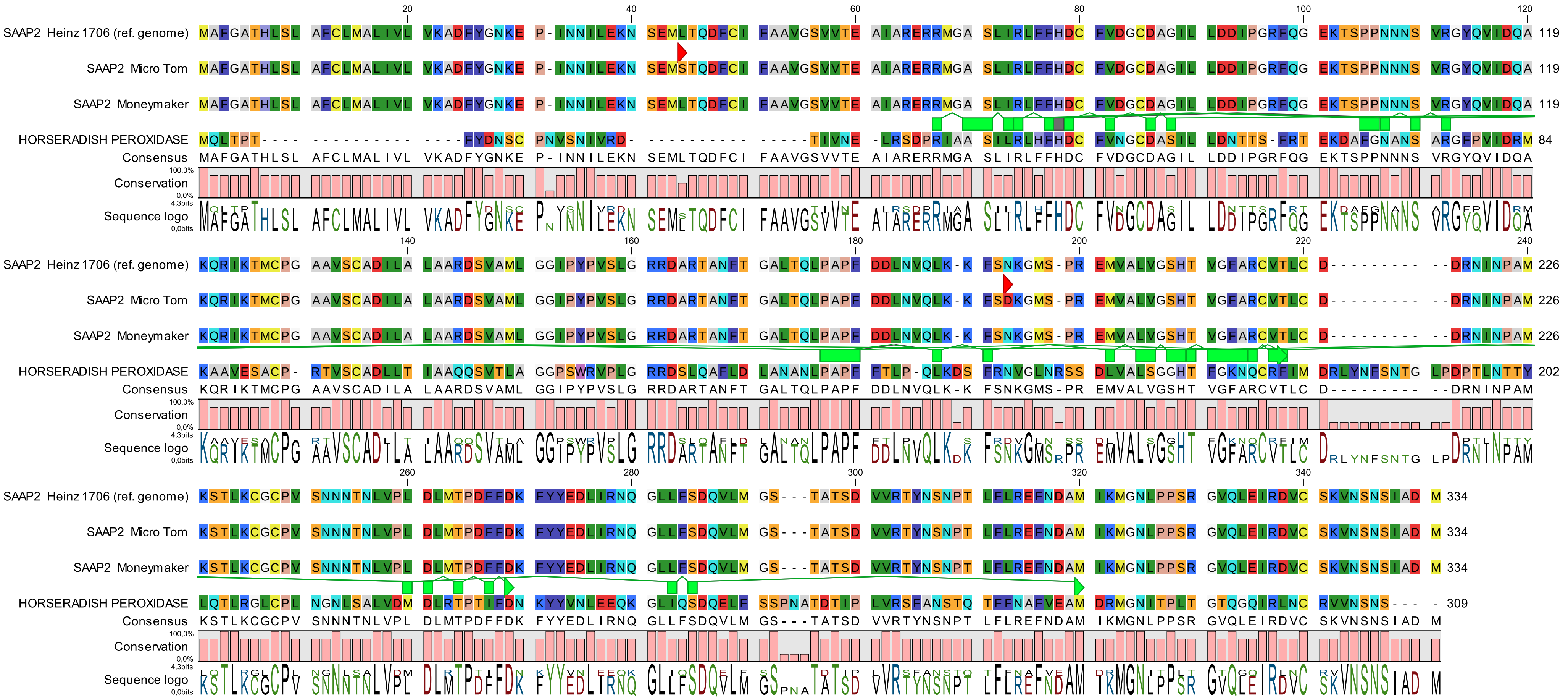

Suppl. Fig. C. SAAP2 amino acid sequences from the reference genome (Heinz 1706 strain) and from the two strains studied in this work, aligned with that of horseradish peroxidase. The two substitutions identified in Micro Tom (red triangles) and the contact positions with the heme (green rectangles) are indicated.
