# Supplementary material for: A new versatile peroxidase with extremophilic traits over-produced in MicroTom cell cultures
Source: Sci Rep. 2023 Sep 15;13:15338. doi: 10.1038/s41598-023-42597-x (PMC10504257; doi:10.1038/s41598-023-42597-x)
Supplement: Supplementary file 4 — Supplementary Figure 4. [file 41598_2023_42597_MOESM4_ESM.pdf]

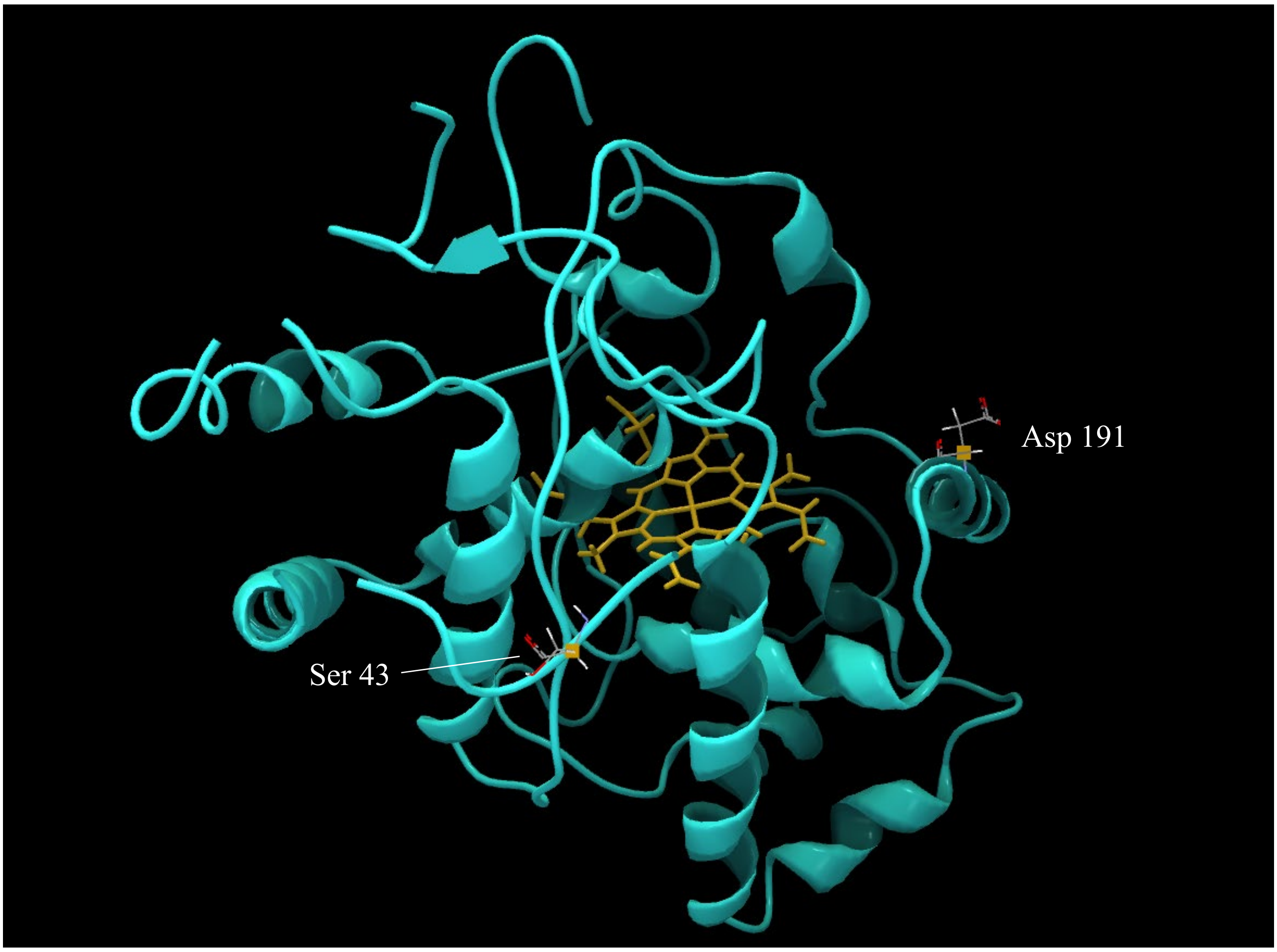

Suppl. Fig. D. 3D model of Micro Tom SAAP2 peroxidase. The heme (in yellow) and the position of the two substituted residues are indicated.
