# Supplementary material for: A new versatile peroxidase with extremophilic traits over-produced in MicroTom cell cultures
Source: Sci Rep. 2023 Sep 15;13:15338. doi: 10.1038/s41598-023-42597-x (PMC10504257; doi:10.1038/s41598-023-42597-x)
Supplement: Supplementary file 5 — Supplementary Information. [file 41598_2023_42597_MOESM5_ESM.pdf]

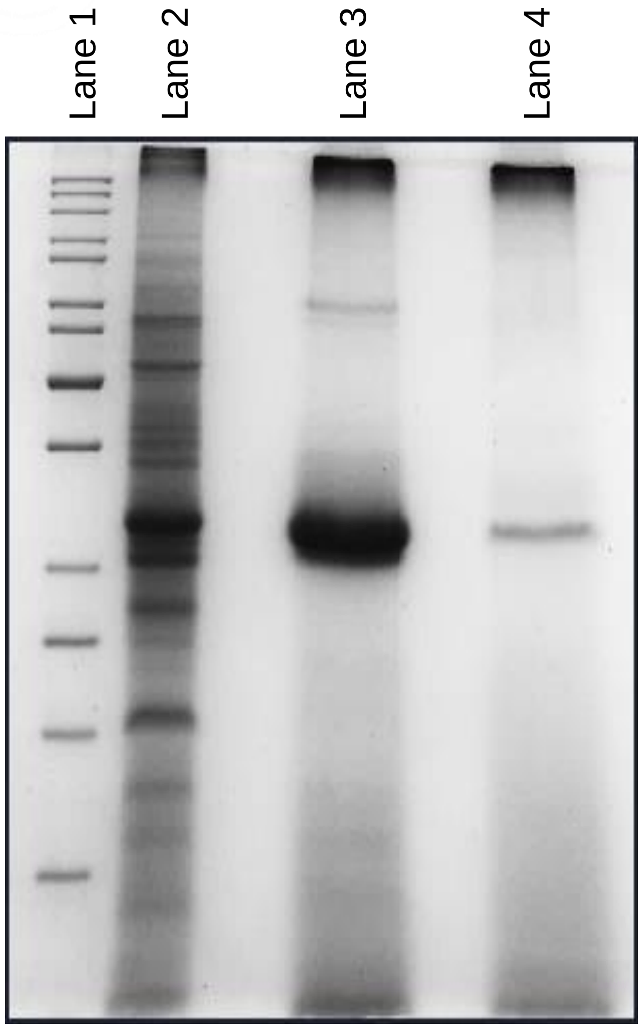

FIG.1 - The gel was cut to remove the lane 4 in which was loaded the total protein extract after thermal treatment at 90°C for 30 min obtained from tomato cell lines.

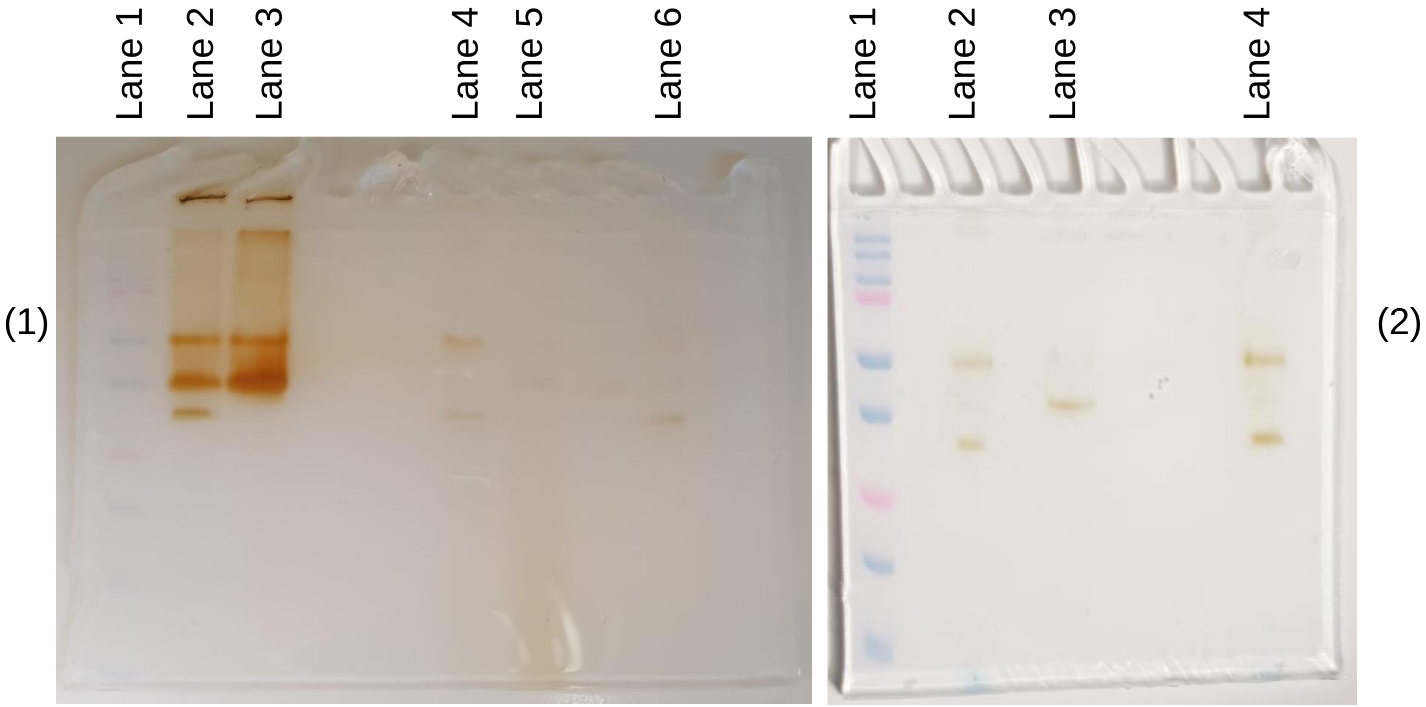

Fig.2 - The activity-stained gels (semi-native PAGE) were cut for different reasons. In the original gel analyzing the Microtom samples (1) were also loaded samples of other tomato cultivars which were not considered in the paper. In the gel 2, samples of crude extracts from MoneyMaker culture cell line were loaded in lane 2 e 4.

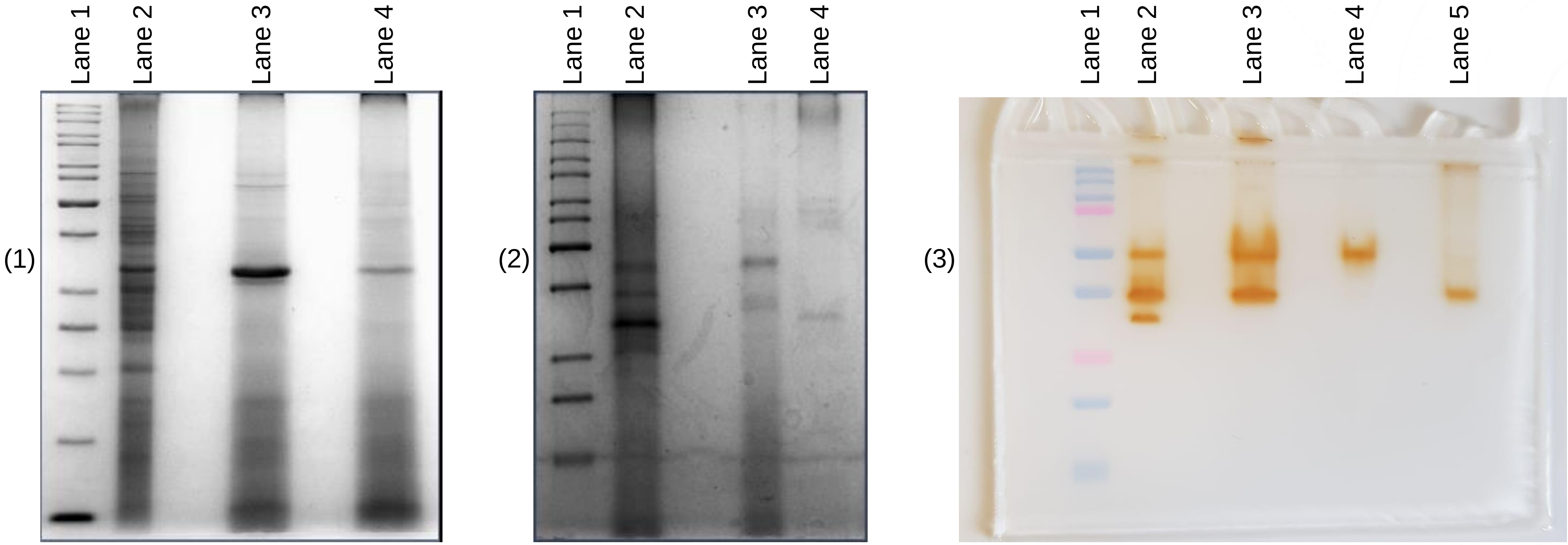

Fig.3 – In the SDS-PAGE reported in the Figure 3A of the paper were shown the lanes 1-3 of the gel 1 and the lanes 3-4 of the gel 2. The gel 3 is the original image of the semi-native PAGE reported in Figure 3B.

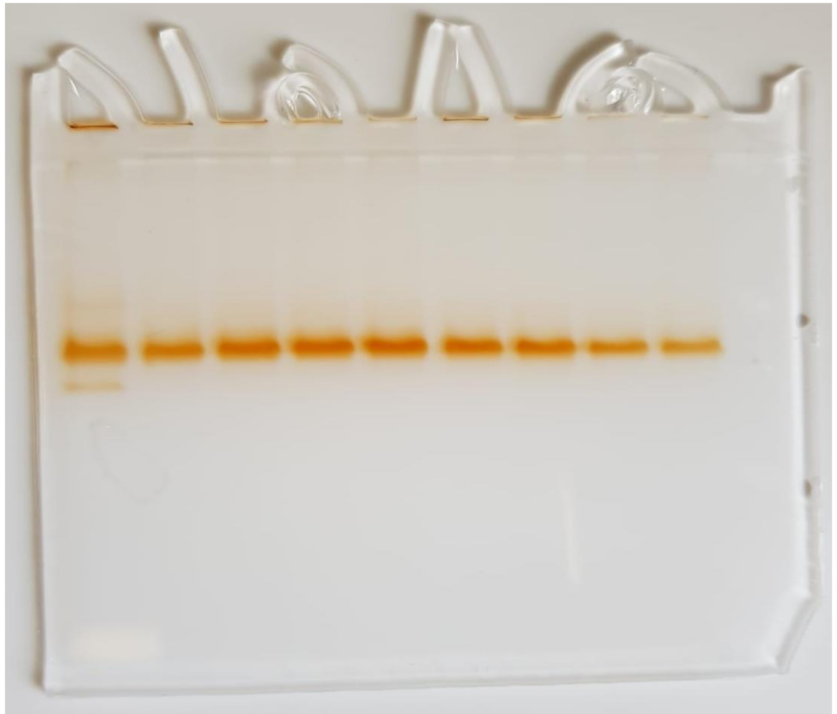

Fig. 5 – Here was reported the original image of the activity-stained gel shown in Figure 5 of the paper concerning the proteolytic resistance of SAAP2 incubated with increasing concentrations of pepsin and at different times.

(1)

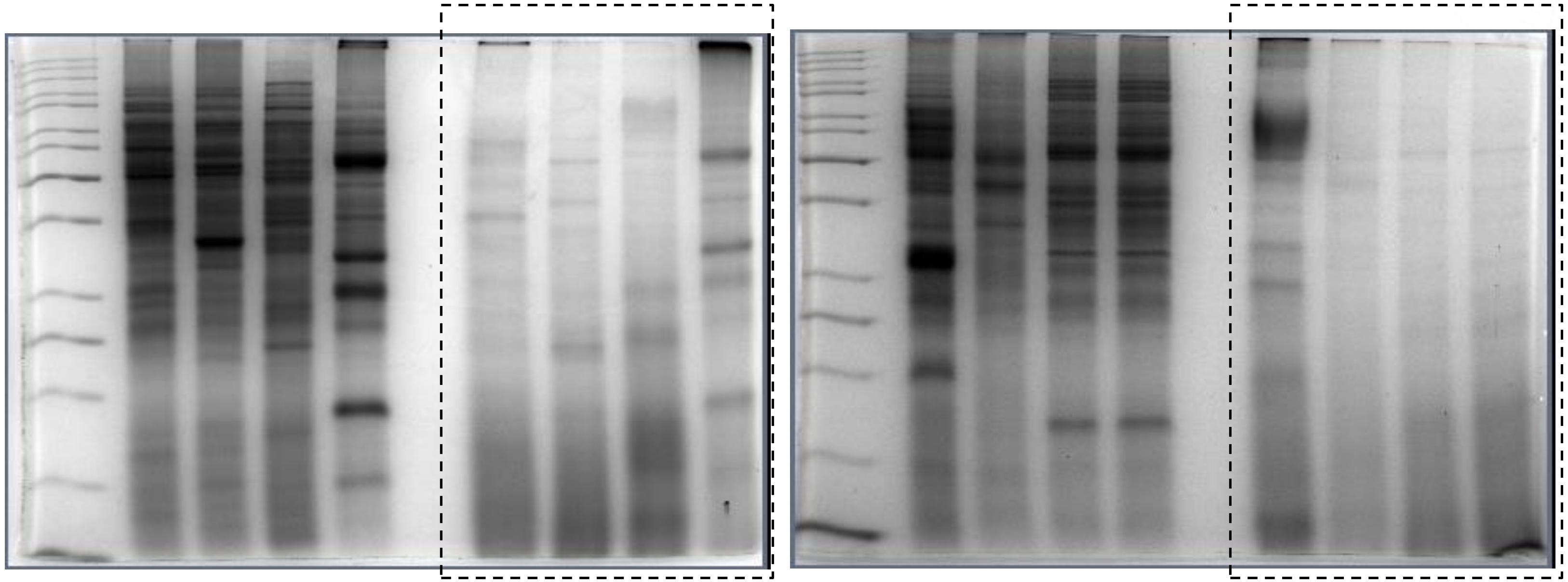

(2)

Fig.6 – In the SDS-PAGE reported in Figure 6A of the paper were shown the two portions evidenced with dotted lines of the gel 1 and 2 regarding the heat treated samples.
